# Preclinical development of an immunoassay for the detection of TREM2: a new biomarker for Alzheimer’s disease

**DOI:** 10.1038/s41598-025-09262-x

**Published:** 2025-07-22

**Authors:** Jie Hu, Huimei Zeng, Jiaqi Lu, Tianpeng Li, Xue Liu, Yang Liu, Weihuan Wen, Weijun Shen, Hongying Chen, Zhicheng Chen

**Affiliations:** 1 College of Life Sciences, Northwest Agriculture and Forestry University, Yangling, 712100 China; 2 Center for Translational Research, Shenzhen Bay Laboratory, Shenzhen, 518132 China

**Keywords:** Alzheimer’s disease, Biomarker, TREM2, Detection, Phenotypic screening, Biomarkers, Alzheimer's disease

## Abstract

Alzheimer’s disease (AD) is a neurodegenerative disorder characterized by the accumulation of amyloid-β (Aβ) plaques and neurofibrillary tangles composed of hyperphosphorylated tau protein. The combination of biomarkers is crucial for AD diagnosis. The triggering receptor expressed on myeloid cells 2 (TREM2), a receptor expressed on microglia, is important in AD pathogenesis. Impairment of TREM2 function aggravates the toxic effects of amyloid plaques, and its activation has been shown to reduce Aβ burden and memory deficits. Increased levels of soluble TREM2 (sTREM2) in blood and cerebrospinal fluid is associated with AD. Therefore, TREM2 could serve as a non-invasive biomarker for AD. In this study, we developed a preclinical immunoassay to detect TREM2 for AD diagnosis. Highly sensitive and specific TREM2 antibodies were produced using the hybridoma technique. The three optimized immunoassays exhibited lower limit of quantitation (LLOQ) of 0.474, 0.807, and 0.415 ng/mL, respectively. These preclinical immunoassays showed high sensitivity and specificity. The sandwich enzyme-linked immunosorbent assay (ELISA) could potentially be used for AD diagnosis.

## Introduction

As of 2023, it is approximated that globally, around 55 million individuals are living with dementia, and nearly 10 million new cases are diagnosed each year^1^. Alzheimer’s disease (AD) is the predominant type of dementia, believed to constitute about 60 to 70 percent of all cases^1^. Early symptoms of AD include forgetting recent events or conversations, which progress to severe memory issues and the inability to perform everyday tasks. There are no therapy to cure AD currently, thus early diagnosis is significant.

AD biomarker detection uses positron emission tomography (PET) imaging, cerebrospinal fluid (CSF), and plasma methods. On one hand, the advent of amyloid-β (Aβ)-PET imaging has enabled noninvasive direct visualization of amyloid beta buildup in living patients exhibiting cognitive deficits, significantly advancing the diagnosis and study of AD. In addition, Tau-PET abnormalities can accurately predict impending cognitive deterioration in both asymptomatic and symptomatic subjects and are sensitive to the regional spread of tau pathology over time^2^. On the other hand, the most validated AD CSF biomarkers are the reduction of Aβ_42_ or Aβ_40_ peptide or total tau (t-tau) protein and the increase in phosphorylated tau (p-tau). These biomarkers are recognized for their diagnostic value in research guidelines and are used clinically across numerous European nations and the United States^3^. While these are traditional diagnosis methods for AD, due to equipment limitation and radioaction of PET imaging method and invasive CSF-based detection approach, they are not suitable for large-scale diagnosis. An optimal biomarker for AD should be reliable, reproducible, and easily collected by noninvasive means. Blood-based biomarkers satisfy these criteria and hold the potential to be used in the diagnosis of AD. However, the accumulation of traditional biomarker tau in cortices such as the temporal and parietal cortices occurs closer to the time of cognitive decline and persists as the disease advances^4–6^. This leaves no time for early diagnosis and intervention. While Aβ begins to accumulate 10–20 years before the onset of cognitive symptoms, with the pace of deposition decelerating around the symptomatic phase, the detection of Aβ in plasma is limited by its low abundance^6^. As a result, its detection would require advanced peptide detection technology, which is not only financially prohibitive and technically complex but also faces difficulties in enhancing the assay precision due to the low concentration of Aβ in plasma.

One prospective strategy is the combination of Aβ peptides and tau protein with other biomarkers in AD pathophysiology and non-AD co-pathology, which is the foundation of the new AD diagnostic criteria at the Alzheimer’s Association International Conference® 2024^7^. The ATN (where A stands for amyloid, T for tau, and N for neurodegeneration) framework aims to stratify individuals within the AD continuum. AD biomarkers can denote the position of an individual on the AD spectrum (state biomarkers). Biomarkers also serve to gauge the progression of AD (stage biomarkers), including tau-PET and biomarkers of neurodegeneration^8^. Diagnostic strategies are currently being refined to suggest the most appropriate combinations.

The use of a combination of multiple biomarkers for diagnostic purposes has been established as a more reliable approach. Evidence suggests that TREM2, a transmembrane protein expressed on myeloid cells and microglia that is essential for enhancing microglial proliferation, phagocytic ability, and survival, is an important factor in the pathogenesis of AD^9^. Rare genetic variants of the *TREM2* gene, such as the R47H variant, have been implicated in increasing the risk of developing AD^10^. The loss of TREM2 function impairs microglial response to amyloid plaques, leading to a toxic cellular state^11^. In addition, interventions such as the stereotactic administration of TREM2 and the activation of TREM2 by adeno-associated virus have demonstrated their ability to decrease amyloid plaque accumulation and reduce functional memory deficits^12^. The extracellular domain (ECD) of TREM2 can be cleaved by ADAM 10/17, becoming soluble TREM2 (sTREM2)^13,14^. In the extracellular environment, sTREM2 is a molecule that can be quantified in both plasma and CSF^15^. Several studies have confirmed that AD patients have higher levels of sTREM2 in their CSF compared to normal individuals^16^. High serum sTREM2 concentration correlates with an increased risk of all-cause dementia, AD, and vascular dementia in the general elderly population^17^. The increase in sTREM2 concentration shows a certain degree of sensitivity and specificity in distinguishing AD patients from healthy individuals, indicating that sTREM2 could serve as a potential biomarker for AD^18^. In addition, elevated plasma TREM2 can be observed as early as the initial phases of AD^19^. The early increase of sTREM2 level in both plasma and CSF suggests its potential use for the early screening of AD^18^. Unlike tau protein and Aβ peptide, plasma concentration of TREM2 is at nanomolar level in early-stage AD and within the detection range of immunoassays. This provides a more reproducible and economical diagnostic method, thus making it an accessible and economically viable option for large-scale early screening of AD.

Given the evidence of TREM2 as a diagnostic biomarker for AD and the need for improved diagnostic tools, we aimed to develop a sensitive and specific immunoassay for the detection of TREM2. Our study focused on producing and screening anti-TREM2 antibodies, developing a sandwich enzyme-linked immunosorbent assay (ELISA) method, and evaluating the performance of the optimized immunoassay in detecting TREM2 protein in AD patients.

## Results

### Development of anti-TREM2 antibodies

**Figure 1:**
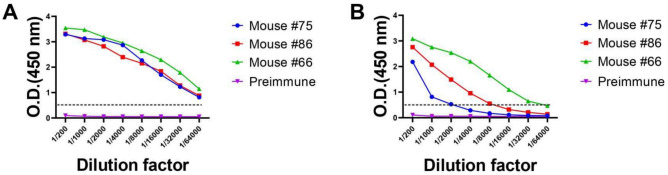
The antiserum titers were determined by indirect ELISA. Serum titers in mice #75, #86, and #66 were tested to measure immune response to TREM2. Serums of preimmune mice were used as negative control. (**A**) Titer of serum against TREM2 ECD Fc was tested by ELISA. (**B**) Titer of serum against TREM2 ECD His was tested by ELISA. The X-axis represented the serum dilution from 1/200 to 1/64,000, and the Y-axis represented the optical density at 450 nm. Error bars equaled ± one standard deviation (SD).

The immunization efficacy was evaluated by measuring the TREM2 antibody titer of immunized mice using indirect ELISA. All samples had optical density (OD) values greater than 0.5 at a dilution ratio exceeding 1/64,000 (Fig. 1A), and for mice #66, the OD value was maintained even at a dilution ratio higher than 1/32,000 (Fig. 1B). The serum titer analysis revealed a strong immune response against TREM2, with mice #66 showing particularly strong reactions. These results suggest that all mice developed a strong immunization against TREM2 antigens.

### Binding affinity and epitope determination of TREM2 monoclonal antibodies to TREM2

**Figure 2:**
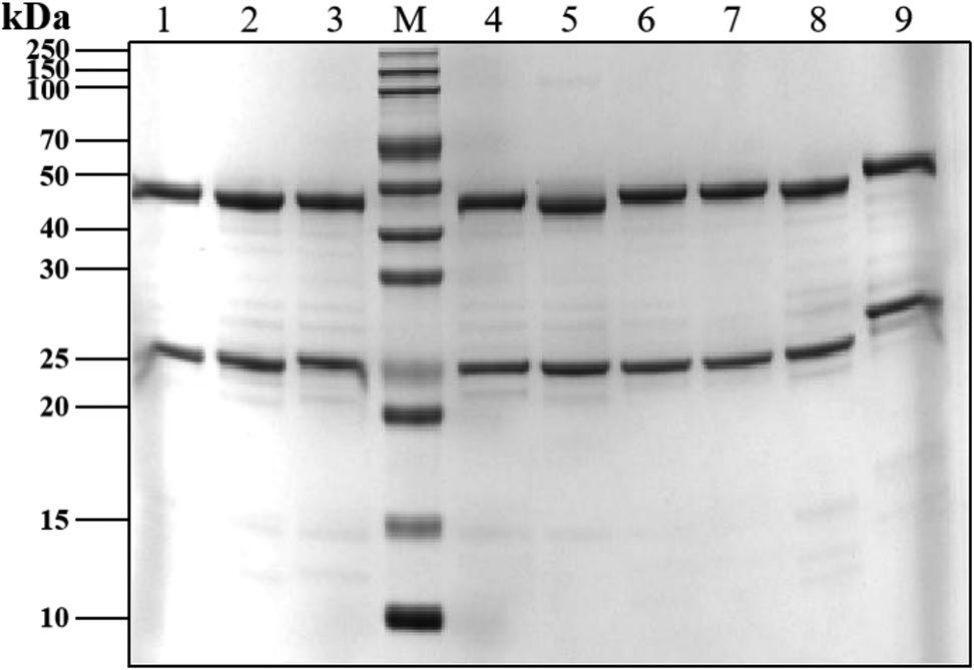
SDS-PAGE analysis of TREM2 antibodies. Lane M: protein marker. Lane 1–9: number corresponded to sample name, 3 ug, reduced. Number 1 corresponded to TREM2#46 mAb; Number 2 corresponded to TREM2#53 mAb; Number 3 corresponded to TREM2#57 mAb; Number 4 corresponded to TREM2#60 mAb; Number 5 corresponded to TREM2#63 mAb; Number 6 corresponded to TREM2#65 mAb; Number 7 corresponded to TREM2#69 mAb; Number 8 corresponded to TREM2#75 mAb; Number 9 corresponded to AL002.

**Figure 3:**
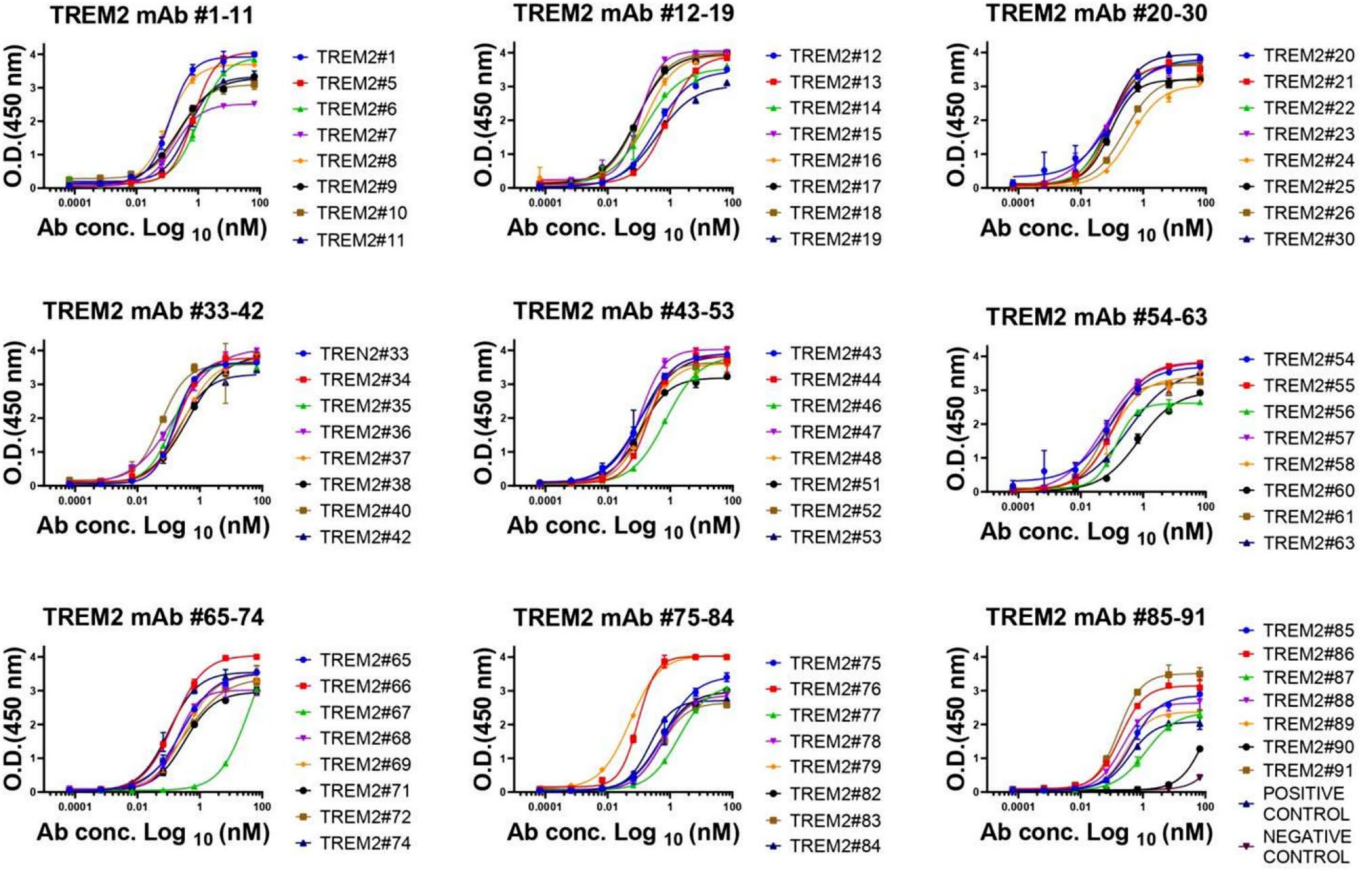
The binding affinity of TREM2 mAbs to antigen TREM2. These mAbs were diluted from 10,000 to 0.001 ng/ml. Error bars equaled ± one standard deviation (SD).

Monoclonal antibodies (mAbs) were purified by protein A column chromatography, followed by sodium dodecyl sulfate–polyacrylamide gel electrophoresis (SDS-PAGE) separation (partly presented in Fig. 2). Full information could be seen in Supplementary Figure S1 and Supplementary Table S1. The binding affinity of anti-TREM2 mAbs to the human TREM2 ECD Fc antigen was evaluated by indirect ELISA (Fig. 3). A total of 64 out of 70 antibodies showed sub-nanomolar half maximal effective concentration (EC50) values, indicating that they bind to the human TREM2 protein (Fig. 3, replications presented in Supplementary Figure S2 and EC50 values presented in Supplementary Table S2).

**Figure 4:**
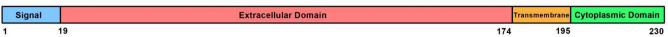
Domains of human TREM2 protein.

**Figure 5:**
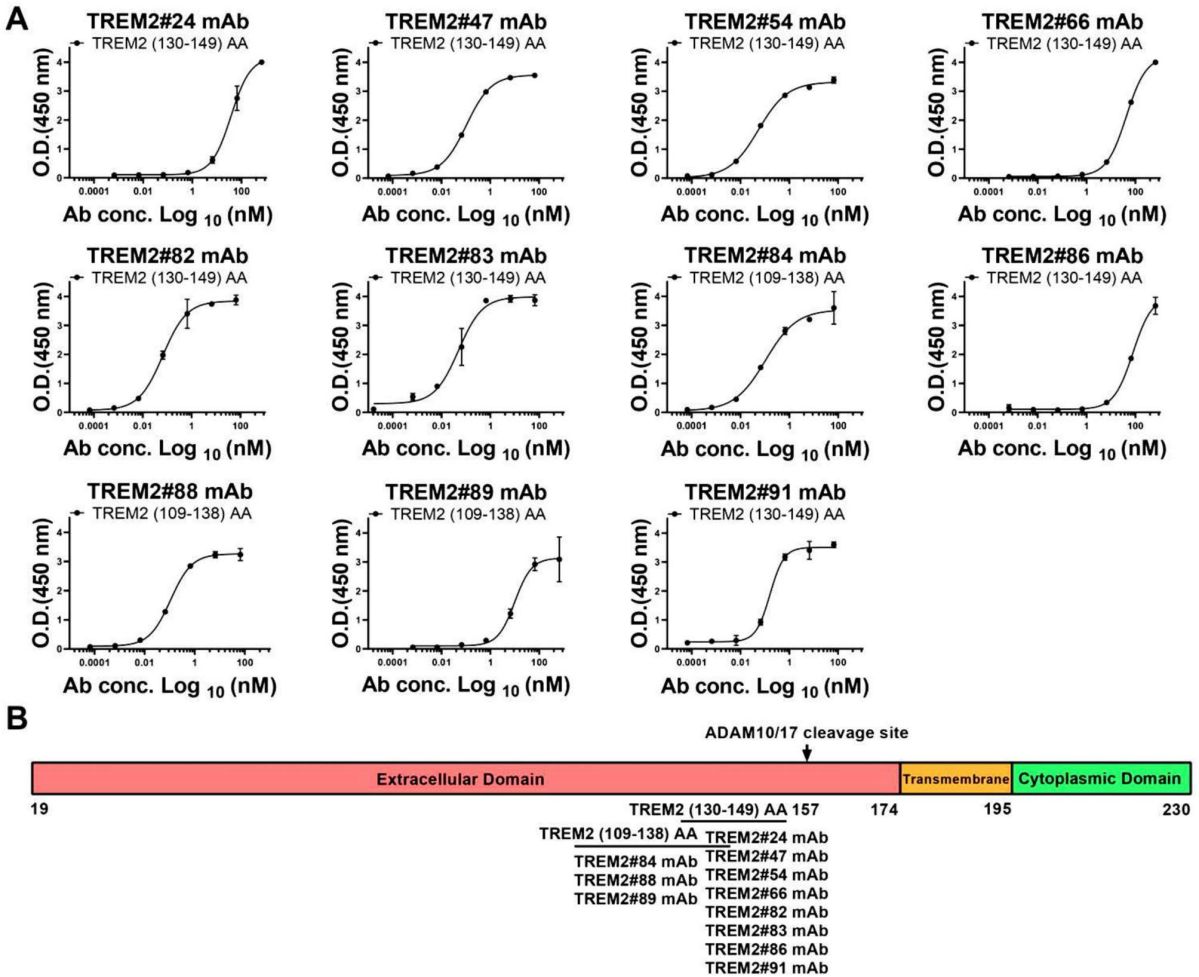
Affinity and Epitope determination of TREM2 mAbs. (**A**) Absorbance was measured from double wells; error bars equaled ± one standard deviation (SD). (**B**) Epitope determination of TREM2 mAbs.

To further examine the epitopes recognized by these mAbs, we synthesized a series of TREM2 peptides, including TREM2 (19–48) amino acid residues (AA), TREM2 (49–78) AA, TREM2 (79–108) AA, TREM2 (109–138) AA, TREM2 (139–174) AA, and TREM2 (130–149) AA conjugated to bovine serum albumin (BSA) (TREM2 (130–149) BSA). The amino acid sequences of these peptides are listed in Table 1, and the domains of the TREM2 protein are shown in Fig. 4. Antibodies TREM2#24, #47, #54, #66, #82, #83, #86, and #91 bound specifically to TREM2 (130–149) BSA, while antibodies TREM2#84, #88, and #89 bound to peptide segment TREM2 (109–138) AA (Fig. 5), suggesting that those antibodies likely bind to different epitopes of TREM2 and they could be potentially used as capture/detection antibody for immunoassay of TREM2.

**Table 1:** The amino acid sequences of peptides.

Peptides	Sequences
TREM2 (19–48) AA	HNTTVFQGVA GQSLQVSCPY DSMKHWGRRK
TREM2 (49–78) AA	AWCRQLGEKG PCQRVVSTHN LWLLSFLRRW
TREM2 (79–108) AA	NGSTAITDDT LGGTLTITLR NLQPHDAGLY
TREM2 (109–138) AA	QCQSLHGSEA DTLRKVLVEV LADPLDHRDA
TREM2 (139–174) AA	GDLWFPGESE SFEDAHVEHS ISRSLLEGEI PFPPTS
TREM2 (130–149) BSA	BSA—CADPLDHRDA GDLWFPGESE S
TREM2 Extracellular Domain (19–174) Fc (TREM2 ECD Fc)	HNTTVFQGVA GQSLQVSCPY DSMKHWGRRK AWCRQLGEKG PCQRVVSTHN LWLLSFLRRW NGSTAITDDT LGGTLTITLR NLQPHDAGLY QCQSLHGSEA DTLRKVLVEV LADPLDHRDA GDLWFPGESE SFEDAHVEHS ISRSLLEGEI PFPPTS—Fc
TREM2 Extracellular Domain (19–174) polyhistidine (TREM2 ECD His)	HNTTVFQGVA GQSLQVSCPY DSMKHWGRRK AWCRQLGEKG PCQRVVSTHN LWLLSFLRRW NGSTAITDDT LGGTLTITLR NLQPHDAGLY QCQSLHGSEA DTLRKVLVEV LADPLDHRDA GDLWFPGESE SFEDAHVEHS ISRSLLEGEI PFPPTS—polyhistidine

TREM2 (130–149) was conjugated to Bovine Serum Albumin (BSA) via a thiol linkage. TREM2 Extracellular Domain (His 19–Ser 174) was linked to human IgG1 Fc tag (Pro 100–Lys 330) at the C-terminus (Cat# TR2-H5254; AcroBiosystems, Beijing, China). TREM2 Extracellular Domain (His 19–Ser 174) was linked to a polyhistidine tag at the C-terminus (Cat# TR2-H52H5; AcroBiosystems).

### Preliminary screening of TREM2 antibody pairs for immunoassays

**Figure 6:**
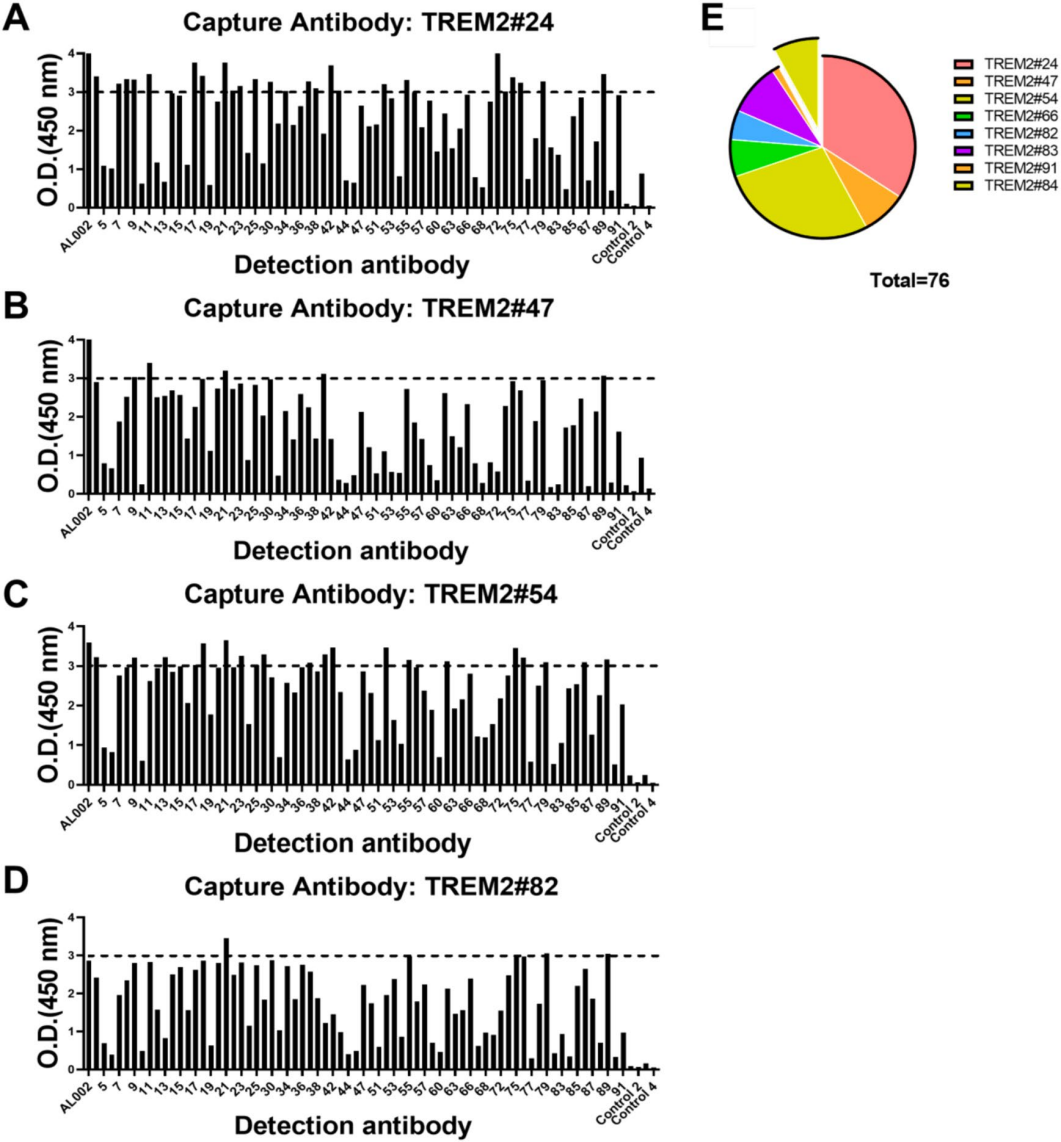
Representative results and a statistical report of TREM2 sandwich ELISA screening. (A-D) Capture antibodies were TREM2#24, #47, #54, #82. A total of 781 antibody pairs were screened, of which 76 antibody pairs showed OD 450 nm values greater than 3.0 ( more information in Supplementary Figure S3). (E) A statistical report detailing the characteristics of the selected antibody pairs was presented. These antibody pairs were methodically categorized by their capture antibodies, which were further subdivided into two distinct clusters based on epitope specificity. The isolated cluster consisted of antibody pairs that featured the capture antibody TREM2 #84, specifically targeting TREM2 (109–138) AA. All remaining antibody pairs recognized TREM2 (130–149) BSA.

To assess the antibody capture and detection performance, we used sandwich ELISA to screen antibody pairs for subsequent optimization. The results of the assays of the 781 antibody pairs, performed at a single concentration, are shown in Fig. 6. Full information of the result could be seen in Supplementary Figures S3 and S4. Out of 781 antibody pairs examined, 76 antibody pairs had OD (450 nm) values greater than 3.0, indicating their high sensitivity.

In the collection of 76 antibody pairs, the predominant character among capture antibodies were their affinity for TREM2 (130–149) BSA. A smaller subset of only 6 pairs showed a preference for binding to the TREM2 (109–138) AA, with the capture antibody in this subset being TREM2#84, as shown in Fig. 6E. As TREM2 (109–138) AA overlaps with immunoglobulin domain (29–112) (Uniprot: Q9NZC2) that has secondary structure for a lot of antibodies to target, there may be overlaps between capture antibodies and detection antibodies in this group, causing lower signal values. Within the group of capture antibodies targeting TREM2 (130–149) BSA, capture antibody #24 stood out for its exceptional compatibility, consistently showing a strong affinity when paired with the majority of detection antibodies, resulting in 26 pairs being selected for further study (Fig. 6E). In conclusion, these antibodies were selected to be further characterized for the identification of the TREM2 recognition.

### Gradient-based sandwich ELISA screening

**Fig. 7:**
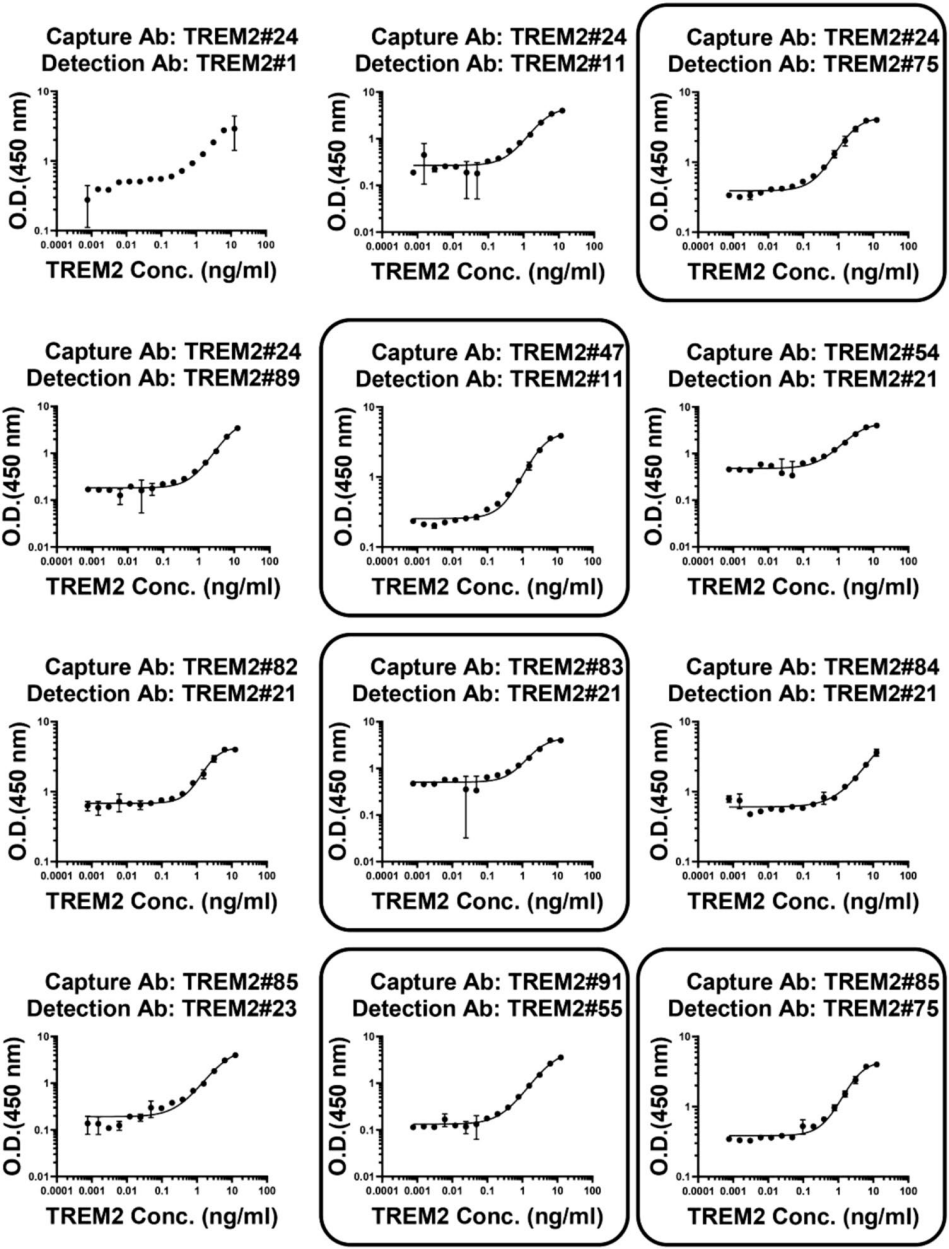
Establishment of TREM2 immunoassays by ELISA. Selected pairs were squared: capture Ab TREM2#24 paired detection Ab TREM2#75, capture Ab TREM2#47 paired detection Ab TREM2#11, capture Ab TREM2#83 paired detection Ab TREM2#21, capture Ab TREM2#85 paired detection Ab TREM2#75, and capture Ab TREM2#91 paired detection Ab TREM2#55. The curve represented the 4PL curve fitting. Error bars equaled ± one standard deviation (SD).

To examine sensitivity and linearity of the immunoassays, statistical analysis was performed using a four-parameter nonlinear logistic regression model to fit the standard curves generated by gradient-based sandwich ELISA screening. From an initial pool of 76 antibody pairs, a total of 12 pairs were selected for outstanding curve fitting after performing an initial gradient-based sandwich ELISA screening to generate the curves (Fig. 7). The selection was further narrowed down based on the sensitivity and the standard curves generated by ELISA screening. The standard curves for the TREM2 antibody pairs detecting the TREM2 ECD Fc are shown in Fig. 7. The following antibody pairs were chosen: TREM2#24 paired with TREM2#75, TREM2#47 paired with TREM2#11, TREM2#83 paired with TREM2#21, TREM2#85 paired with TREM2#75, and TREM2#91 paired with TREM2#55. These five antibody pairs showed high sensitivity and could be developed as diagnostic tools.

### Validation of antibody pairs

To refine the antibody pair selection and evaluate the assay performance, the immunoassay was optimized. This experiment aimed at determining the lower limit of quantitation (LLOQ), range and coefficient of determination (R^2^) for each antibody pair. The concentrations of both capture and detection antibodies were reduced to 0.5 µg/mL to minimize background OD values. The standard curves were constructed for the TREM2 antibody pairs with the TREM2 protein.

**Fig. 8:**
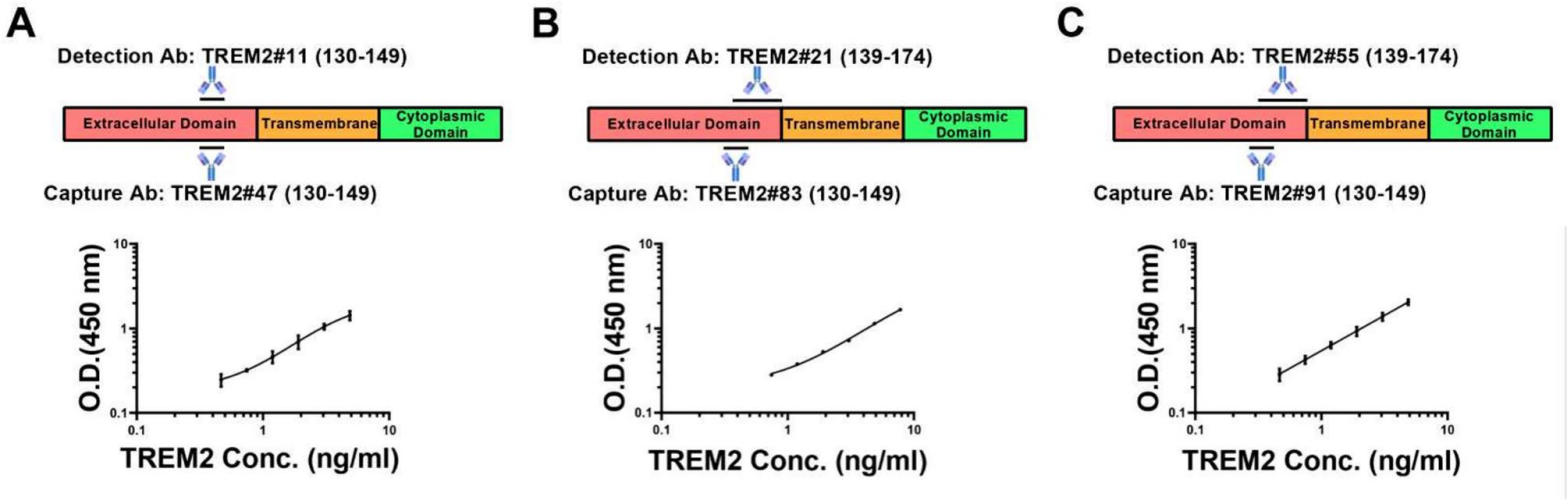
Standard curves of three immunoassays. Selected pairs: capture Ab TREM2#91 paired detection Ab TREM2#55, capture Ab TREM2#83 paired detection Ab TREM2#21, and capture Ab TREM2#47 paired detection Ab TREM2#11. The binding sites of three pairs were different. Error bars equaled ± one standard deviation (SD).

The selected pairs included the following combinations: capture antibody TREM2#91 paired with detection antibody TREM2#55, capture antibody TREM2#83 paired with detection antibody TREM2#21, and capture antibody TREM2#47 paired with detection antibody TREM2#11. The assays were consistent across duplicates, indicating reliable assay performance (Supplementary Figure S5). The optimized antibody pairs satisfied three criteria that LLOQ values below 0.810 ng/mL, R^2^ values higher than 0.997, and range length greater than 4.400 ng/mL, confirming their suitability for further use in TREM2 detection assays (Table 2). Antibody TREM2#47 was found to bind specifically to TREM2 (130–149) BSA, as shown in Fig. 8A. Antibody TREM2#11 recognized an epitope in peptide segment TREM2 (139–174) AA. Antibody TREM2#83 recognized an epitope in the TREM2 (130–149) BSA, as shown in Fig. 8B. In addition, antibody TREM2#21 was found to bind to the TREM2 (139–174) AA. Similarly, antibody TREM2#91 recognized an epitope mapped to the TREM2 (130–149) BSA (Fig. 8C), while TREM2#55 bound to the peptide TREM2 (139–174) AA. These three selected antibody pairs had distinct binding sites in the TREM2 protein, which indicates a broad range of targets for detection.

**Table 2:** Sandwich ELISA was performed two times for each pair.

Antibody pairs	Capture Ab: TREM2#47 Detection Ab: TREM2#11			Capture Ab: TREM2#83 Detection Ab: TREM2#21			Capture Ab: TREM2#91 Detection Ab: TREM2#55		
Parameters	LLOQ (ng/mL)	Range (ng/mL)	R^2^	LLOQ (ng/mL)	Range (ng/mL)	R^2^	LLOQ (ng/mL)	Range (ng/mL)	R^2^
Repeat 1	0.474	0.474–4.883	0.996	0.807	0.807–7.813	0.999	0.415	0.415–4.883	0.999
Repeat 2	0.182	0.182–4.883	0.999	0.388	0.388–7.813	0.998	0.347	0.347–4.883	0.997
Average	0.328	–	–	0.598	–	–	0.381	–	–

These pairs satisfy three screening criteria: LLOQ values below 0.810 ng/mL, R^2^ values higher than 0.997, and range length greater than 4.400 ng/mL.

## Discussion

According to the latest revised criteria for the diagnosis and staging of Alzheimer’s disease in 2024 released by the National Institute of Aging and the Alzheimer’s Association (NIA-AA), accurate plasma assays targeting individual Aβ_42_, Aβ_42_/Aβ_40_ ratio, p-tau181, p-tau217, and Aβ_42_/Aβ_40_ ratio in combination with p-tau181 or p-tau217 can be used for the diagnosis of AD^20^. Aβ42/Aβ40 ratio performs better than Aβ42 alone in distinguishing AD pathology from non-AD pathology^21^. Plasma biomarker Aβ42/Aβ40 ratio changes decades before brain amyloid accumulation, making it suitable for early diagnosis^22^. The diagnostic performance of blood p-tau217 for AD is superior to that of p-tau181 and other biomarkers like Aβ42/Aβ40^23^. P-tau217 can predict the occurrence of cognitive impairment 10 years in advance, making it a good biomarker for the early diagnosis of AD^24^. However, the accuracy of test results for Aβ42/Aβ40, p-tau217, and their combined diagnosis is limited by the standardization of the detection platform, and there are significant differences in the reference ranges between different detection platforms^25^. Comparably, the TREM2 immunoassay method is based on a mature sandwich ELISA platform, which has higher detection accuracy. Additionally, glial fibrillary acidic protein and neurofilament light have been explored as biomarkers in AD clinical trials. Elevated glial fibrillary acidic protein levels with a high increase rate are observed in females, especially those who are premenopausal, which suggests the potential utility of glial fibrillary acidic protein as a gender-specific biomarker for predicting dementia risk in women^26^. The increase in blood neurofilament light levels can be observed as much as 16 years prior to the appearance of AD symptoms and it predicts disease progression and brain neurodegeneration in the early, presymptomatic stages of familial Alzheimer’s disease^27^. Similarly to TREM2, neurofilament light is not specific for AD, thus unlikely to be used alone for diagnosis of AD^28^. In a research combining neurofilament light and other biomarkers for diagnosis of AD, the risk of dementia-specific death was significantly elevated in individuals with high serum concentrations of both neurofilament light and glial fibrillary acidic protein, compared to those with low levels of both biomarkers^29^. However, plasma concentrations of neurofilament light and glial fibrillary acidic protein are at picomolar level, much lower than that of TREM2, which makes it hard to be detected by simple methods^30^. Regarding existing TREM2 assays, Human TREM2 gene R47H mutation Real Time PCR Kits (www.creative-biogene.com) are used for AD diagnosis. However, compared to TREM2 protein detection, the cost is higher and it is not suitable for large-scale screening. A homogeneous AlphaLISA Human TREM2 Detection Kit from Revvity offers simple operation and is suitable for high-throughput detection, which could potentially be developed into an AD diagnostic method. However, the homogeneous system has high background signals, which may lead to false-positive or false-negative results. Compared to sandwich ELISA, homogeneous immunoassay requires more demanding experimental conditions. A novel study that aimed to discover prior combinations of AD biomarkers developed six highly sensitive immunoassays detecting Tau(1–22)-pTau231, Tau(1–22)-pTau217, and Tau(1–22)-pTau181 on ultra-sensitive simoa platform with LOD at picomolar level^31^. In addition, a study has identified 19 key ‘hub proteins’ within the novel blood biomarkers for AD, which exhibits stage-specific changes and have the ability to differentiate between the diverse stages of AD^32^. This highlights the promising future of researches that focus on identifying optimal combinations of blood biomarkers for AD.

Plasma diagnosis has the advantages of convenient collection, suitable for large-scale screening and long-term monitoring, so it has attracted more and more attention in recent years. Our TREM2 immunoassay is characterized by high sensitivity, repeatability and affordable budget. However, TREM2 is not only expressed on microglia, but also on macrophage. It plays a pivotal role in diseases including neurodegenerative disorders, inflammation, cancer, metabolic diseases, and cardiac conditions. As TREM2 detection is not AD-specific, combination of multiple biomarkers can be used for diagnosis of AD, and the diagnostic performance is better than single biomarker. Furthermore, the concentration of plasma TREM2 is approximately nanomolar, compared with pTau217 at approximately picomolar. The current clinical methodology can be applied to AD diagnosis rather than acquiring a single-molecule detection machine. Therefore, the development of the immunoassay for TREM2 requires several steps in the following clinical stage. Firstly, it is crucial to compare our TREM2 immunoassay method with established methods included in the AD diagnostic criteria such as CSF detection or PET imaging. Since cerebral Aβ deposition is a characteristic of AD pathogenesis, predicting brain Aβ deposition is key for AD diagnosis. One study compared the correlation between blood biomarkers and brain Aβ deposition^33^. Secondly, our lab is also working on developing a plasma p-Tau immunoassay for AD diagnosis^31^. Plasma p-tau181, p-tau217, and p-tau231 can relatively better detect pathological changes between individuals with normal cognition and those with mild cognitive impairment^34^. Additionally, a comprehensive study will be initiated that integrates numerous core biomarkers for diagnosis of AD. A combination of plasma p-tau and Aβ_42_/Aβ_40_, particularly high-performing Aβ_42_/Aβ_40_ assays, was found to be the most effective biomarker combination for detecting amyloid pathology in cognitively unimpaired individuals^35^. However, the complexity of sample pretreatment, the difficulty of mass spectrometry data analysis, and the high costs associated with purchasing and maintaining mass spectrometers make mass spectrometry detection of Aβ_42_/Aβ_40_ unsuitable for early screening of AD. Therefore, it is necessary to develop new combinations of blood biomarkers like TREM2 and p-Tau to ensure the accuracy and simplicity of detection.

The sandwich immunoassay is known for its higher specificity, as it requires antibodies to recognize two distinct epitopes on a protein antigen. After antibody purification, an initial indirect ELISA was performed to confirm that the antibody binding to human TREM2 was dose-dependent and to mitigate non-specific bindings. This validation step was critical for the selection of suitable antibody pairs for the subsequent sandwich ELISA. The indirect ELISA conducted before the sandwich ELISA was instrumental in precluding false negative outcomes. Moreover, the occurrence of negative results in the sandwich ELISA indicates that the antibody pairs may be recognizing the same epitope on the antigen.

The shedding of sTREM2 may have a major role in AD pathogenesis. sTREM2 triggers the release of pro-inflammatory cytokines via the NF-κB signaling pathway and enhances microglial viability by activating the Akt/GSK3β/β-catenin signaling pathway in primary microglia^36^. On the other hand, sTREM2 has been shown to facilitate the removal of amyloid plaques in a microglia-dependent manner, as evidenced by the findings of a study in a mouse model^37^, suggesting a protective role in AD. It has been proposed that sTREM2 could act as an extracellular signal that binds to synapse surface receptors, prompting microglial involvement in the elimination of excessive synapses and shaping the appropriate brain connectivity^38^. By regulating synaptic pruning, sTREM2 may be implicated in interneuronal signaling. Moreover, sTREM2 can directly interact with pathogenic entities, such as Aβ, or indirectly regulate inflammation^39^. In this study, we measured sTREM2 levels, and thus this method can be used to investigate the mechanism of action of sTREM2 in AD and the role of sTREM2 shedding at the microglial surface in the progression of AD.

A previous study suggested that elevated serum sTREM2 levels could serve as an indicator for predicting cognitive decline in patients with inadequately managed type 2 diabetes, independent of obesity^40^. However, another study focusing on type 2 diabetic patients and their cognitive status found a decrease in serum sTREM2 levels^41^. Also, a pan-cancer study found that TREM2 had distinct expression patterns in tumor *versus* normal tissues^42^. Additionally, TREM2 expression has been linked to tumor mutational burden, microsatellite instability, and the infiltration of immune cells across multiple cancer types. For example, TREM2^high^ multinucleated giant cells significantly contribute to a favorable prognosis in both untreated and preoperatively managed squamous cell carcinoma, and tracking these cells will greatly enhance the clinical care of patients afflicted with head and neck squamous cell carcinoma^43^. These findings highlight the intricate nature of TREM2 and its promising potential as both a biomarker and a therapeutic target.

In conclusion, this study successfully developed a sensitive and specific immunoassay for the detection of TREM2 protein levels, enabling the accurate measurement of TREM2 levels in AD patients. Going beyond this study, further validation of the immunoassay for TREM2 is required. Firstly, plasma detection results should be further confirmed by CSF detection or PET imaging. Then a comprehensive project combining numerous core biomarkers for diagnosis of AD will be put forward.

## Materials and methods

### Materials and reagents

Peptides were synthesized from the human TREM2 protein sequence by Sangon Biotech Co., Ltd. (Shanghai, China), and named according to their position within the TREM2 protein structure (Fig. 4). TREM2 Peptide (130–149) was conjugated to BSA by a thiol linkage. The TREM2 ECD (His 19-Ser 174) was linked to human IgG1 Fc tag (Pro 100-Lys 330) at the C-terminus (Cat# TR2-H5254; AcroBiosystems, Beijing, China). The TREM2 ECD (His 19- Ser 174) was linked to a polyhistidine tag at the C-terminus (Cat# TR2-H52H5; AcroBiosystems) (Table 1). This study is performed in accordance with relevant guidelines and regulations. All methods are reported in accordance with ARRIVE (Animal Research: Reporting of In Vivo Experiments) guidelines.

### Immunization and ethics statement

Five 6-week-old male BALB/C mice (weighing 18–22 g) were purchased from Zhuhai BesTest Bio-Tech Co., Ltd. (Zhuhai, China). BALB/C mice were chosen to be immune animal because all the mouse myeloma cell lines used for the hybridoma technology were derived from them. The mice were housed in a climate-controlled environment (temperature range of 20–26 °C, with a 12-h light/dark cycle) at the Shenzhen Bay Laboratory (Shenzhen, China). The care and welfare of the animals were in compliance with the guidelines and regulations set forth by the National Institutes of Health (Bethesda, MD, USA). The mice were randomly assigned to different groups to minimize bias. The researchers performing the immunizations and subsequent analysis were blinded to the treatment groups to reduce subjective bias. These practices ensure that our study meets the high standards set by the ARRIVE guidelines for the ethical and responsible conduct of animal research. BALB/C mice were first immunized with complete Freund’s adjuvant. The antigen used for the primary immunization was a fusion protein of human TREM2 ECD with an Fc tag (TREM2 ECD Fc), at a dose of 100 μg per mouse. The mice received two immunization boosts with TREM2. After the final immunization, mice were euthanized with an overdose of carbon dioxide. A secondary method of euthanasia, cervical dislocation, was used to confirm death. All experiments were performed in accordance with ARRIVE guidelines. The animal study was approved by the Animal Ethics Committee of the Shenzhen Bay Laboratory (agreement code: AECZC202101, December 2021). The spleen cells from these mice were fused for hybridoma generation.

Indirect ELISA was performed to evaluate antiserum titers. Initially, we coated the uncoated Nunc™ MaxiSorp™ 96-well ELISA Plates (Cat# 423501; BioLegend, San Diego, CA, USA) with 1 μg/mL (100 μL/well) human TREM2 ECD Fc or His at 4 °C, overnight. Afterwards, after washing the wells four times with washing buffer (T-PBS, PBS plus 0.05% Tween 20, pH 7.4), the wells were blocked with 2% BSA in washing buffer at 37 °C for 1.5 h. After washing the wells, the serum was serially diluted from 1:200 to 1:64,000 with eight gradients, and the diluted serum samples were added to each well and the plate was incubated at 37 °C for 1 h. Serums of preimmune mice were used as negative control. Subsequently, after washing the wells again, the diluted horseradish peroxidase (HRP)-conjugated rabbit anti-mouse IgG F(ab′)_2_ secondary antibody (Cat# 31451; Invitrogen, Carlsbad, CA, USA) was added to each well, and the plate was incubated at 37 °C for 1 h. Then, after washing the wells, the 3,3′,5,5′-tetramethylbenzidine (TMB) substrate solution (Cat# PR1200; Solarbio, Beijing, China) was added to each well, and the plate was incubated at 37 °C for 5 min in the dark. The reaction was terminated by adding 50 μL/well of ELISA stop solution (Cat# C1058; Solarbio). The absorbance was subsequently measured at 450 nm with a versatile microplate reader (BioTek Instruments, Inc., Vermont, USA). Statistics were analyzed when signal outputs (OD at 450 nm) from each sample were linked by a broken line according to a series of dilution factors.

### Hybridoma generation and antibody purification

#### Cell fusion

SP2/0 cells were obtained from Shenzhen TOP Biotechnology Co., Ltd. (Shenzhen, China). The SP2/0 cells were thawed and subcultured. The hypoxanthine-aminopterin-thymidine (HAT) selection medium was prepared with Dulbecco’s modified Eagle’s medium (DMEM), 50X HAT (Cat# 21060017; Thermo Fisher Scientific Inc., Waltham, MA, USA), and fetal bovine serum (FBS). Feeder cells from three Kunming (KM) mice were plated. Spleens from BALB/C mice were processed to obtain a B cell suspension, which was centrifuged, resuspended in DMEM, and enriched for plasma cells using the EasySep™ Mouse CD138 Positive Selection Kit (Cat# 18957; Stemcell Technologies, Vancouver, BC, Canada). Cells were gently tapped and incubated on ice before fusion with polyethylene glycol (PEG) in a water bath at 37 °C. After fusion, cells were diluted with DMEM high-glucose medium, centrifuged, and resuspended in complete HAT medium. The suspension was added to 96-well plates with feeder cells and cultured in an incubator with 5% CO_2_, at 37 °C.

#### Clone of hybridoma cells

Hybridoma cells were cultured in HAT medium in an incubator at 37 °C with 5% CO_2_ for one week. Subsequently, the cell culture was supplemented with 100 µL of sodium hypoxanthine and thymidine (HT) medium (Cat# 11067030; Thermo Fisher Scientific Inc.). The hybridomas producing mAbs against TREM2 were identified by ELISA. The remaining positive fusion cells were supplemented with 100 µL of HT medium. Positive hybridoma cells were then subjected to three rounds of sub-cloning and cultured in HT medium. Hybridomas were sequenced to determine the antibody DNA before cryopreservation.

#### Production of mAb

Affinity chromatography was performed using a MabSelect Xtra gravity column (Cat# 17526904, Cat# 17043501; Cytiva, Little Chalfont, UK) to purify the mouse antibodies according to the manufacturer’s instructions. For the purity analysis of the purified mAbs, 3 μg of each mAb was loaded onto a 10% SDS-PAGE gel. Electrophoresis was performed at 100 V for one hour, followed by Coomassie blue staining of the gel for three hours at room temperature on a shaker to visualize the separated proteins. After Coomassie blue staining, the gel was decolored, and images were captured using the GenoSens 2100 Touch imaging system (Clinx Science Instruments Co., Ltd., Shanghai, China).

### Antibody affinity verification and epitope determination

The affinity of antibodies to the human TREM2 ECD and TREM2 peptides was assessed by ELISA, which was performed three times with an irrelevant protein used as negative control. The ELISA was performed using Nunc™ MaxiSorp™ ELISA 96-well Plates (Cat# 423501; BioLegend) coated by incubating the plate at 4 °C overnight with 1 μg/mL of the TREM2 protein in 100 μL of Coating Buffer (Cat# C1055; Solarbio) per well. Subsequently, after washing the plate four times with 300 μL of washing buffer, and blocking the wells with 2% BSA, 100 μL of the diluted primary antibody was added and the plate was incubated at 37 °C for one hour with concentrations ranging from either 10,000 or 100,000 ng/mL, ten fold gradient dilution, seven gradients and two replications. Blank control was performed without adding primary antibody. AL002, an identified anti-TREM2 antibody, and an irrelevant antibody were used as positive and negative control, respectively. Then, after washing the plate, 100 μL of a 1:10,000 diluted solution of HRP-conjugated rabbit anti-mouse IgG F(ab′)_2_ secondary antibody (Cat# 31451; Invitrogen), was added into each well and the plate was incubated at 37 °C for one hour. Finally, after washing the plate, 100 μL of TMB substrate solution (Solarbio, Cat# PR1200) was added into each well and the plate was incubated for five minutes before adding the ELISA stop solution (Cat# C1058; Solarbio). The absorbance was subsequently quantified at 450 nm with a versatile microplate reader (BioTek Instruments, Inc., Vermont, USA). Curves were fitted using a four-parameter nonlinear logistic regression model by correlating the signal output (OD at 450 nm) with the logarithm of the analyte’s concentration. EC50 was calculated to reflect sensitivity and affinity of the antibody.

### Antibody screening by sandwich ELISA

The ELISA plate was coated with 100 μL capture antibody in coating buffer and then incubated overnight at 4 °C. The sandwich ELISA screening process was performed with two replications. After washing, the plate was blocked with 2% BSA in washing buffer at 37 °C for 1.5 h, followed by another washing step. Then, 100 μL of diluted human TREM2 ECD Fc (from 12.5 ng/mL to 0.000763 ng/mL, 1.6 fold gradient dilution) was added and incubated at 37 °C for 1 h. After another washing step, 100 μL of a 2 μg/mL biotinylated detection antibody (Cat# A35358; Thermo Fisher Scientific Inc.; the antibody was conjugated to biotin following the manufacturer’s instructions) was added into each well and the plate was incubated at 37 °C for 1 h. Control one was performed without adding TREM2 ECD Fc. Control two was performed without adding TREM2 ECD Fc and detection antibody. Streptavidin-HRP (Cat# ab7403; Abcam, Cambridge, UK), diluted 1:10,000, was then added into each well and the plate was incubated for an hour at 37 °C. After washing, substrate solution was added, and the plate was incubated in the dark at 37 °C for 10 min. After terminating the reaction by adding the ELISA stop solution (Cat# C1058; Solarbio), the final OD values were measured at 450 nm. Standard curves were fitted using a four-parameter nonlinear logistic regression model by plotting the logarithm of the signal output (OD at 450 nm) against the logarithm of the analyte’s concentration. Characteristics of TREM2 immunoassay were obtained. Lower limit of quantitation (LLOQ) is determined by calculating the lowest concentration at which the CV is less than 20%. Range is calculated from the LLOQ to either the top concentration or the ULOQ, whichever is smaller. Coefficient of determination (R^2^) is defined by the equation R^2^ = 1-Residual Sum of Squares/Total Sum of Squares. There are three screening criteria: LLOQ values below 0.810 ng/mL, R^2^ values higher than 0.997, and range length greater than 4.400 ng/mL. Plasma concentration of TREM2 is at nanomolar level, within the detection range according to our criteria, and can be accurately measured by our immunoassays.

### Statistical analysis

For the analysis of indirect ELISA data, a four-parameter nonlinear logistic regression model was used. The model is defined by the following equation Y = Bottom + (Top–Bottom)/(1 + 10^((LogEC50-X) * HillSlope)). Standard curves generated by indirect ELISA were fitted using the four-parameter nonlinear logistic regression model by correlating the signal output (OD at 450 nm) with the logarithm of the concentration of the analyte. The standard curves generated by sandwich ELISA were fitted by the four-parameter nonlinear logistic regression model by plotting the logarithm of the signal output (OD at 450 nm) against the logarithm of the the concentration of the analyte.

## Supplementary Information

Supplementary Information 1.

Supplementary Information 2.

Supplementary Information 3.

## Data Availability

All data generated or analysed during this study are included in this published article [and its supplementary information files].
