# Supplementary material for: Preclinical development of an immunoassay for the detection of TREM2: a new biomarker for Alzheimer’s disease
Source: Sci Rep. 2025 Jul 22;15:26525. doi: 10.1038/s41598-025-09262-x (PMC12280022; doi:10.1038/s41598-025-09262-x)
Supplement: Supplementary file 2 — Supplementary Information 2. [file 41598_2025_9262_MOESM2_ESM.pdf]

Immunoassay Development for TREM2: A New Biomarker for Alzheimer's Disease Diagnosis

Jie Hu 1, Huimei Zeng 2, Jiaqi Lu 2, Tianpeng Li 2, Xue Liu 2, Yang Liu 2, Weihuan Wen 2, Weijun Shen 2,*, Hongying Chen 1,*, Zhicheng Chen 2,*

1 School of Life Sciences, Northwest Agriculture and Forestry University, Yangling, 712100, China.
2 Center for Translational Research, Shenzhen Bay Laboratory, Shenzhen 518132, China.

* To whom the correspondence should be addressed;
Hongying Chen,
School of Life Sciences, Northwest Agriculture and Forestry University, Yangling, 712100, China.
Email: chenhy@nwsuaf.edu.cn (Hongying Chen);
Zhicheng Chen,
Center for Translational Research, Shenzhen Bay Laboratory, Shenzhen 518132, China.
Email: chenzc@szbl.ac.cn (Zhicheng Chen);

sFigure 1 SDS-PAGE analysis of TREM2 antibodies

sTable 1 SDS-PAGE of TREM2 antibodies

TREM2 mAb SDS-PAGE

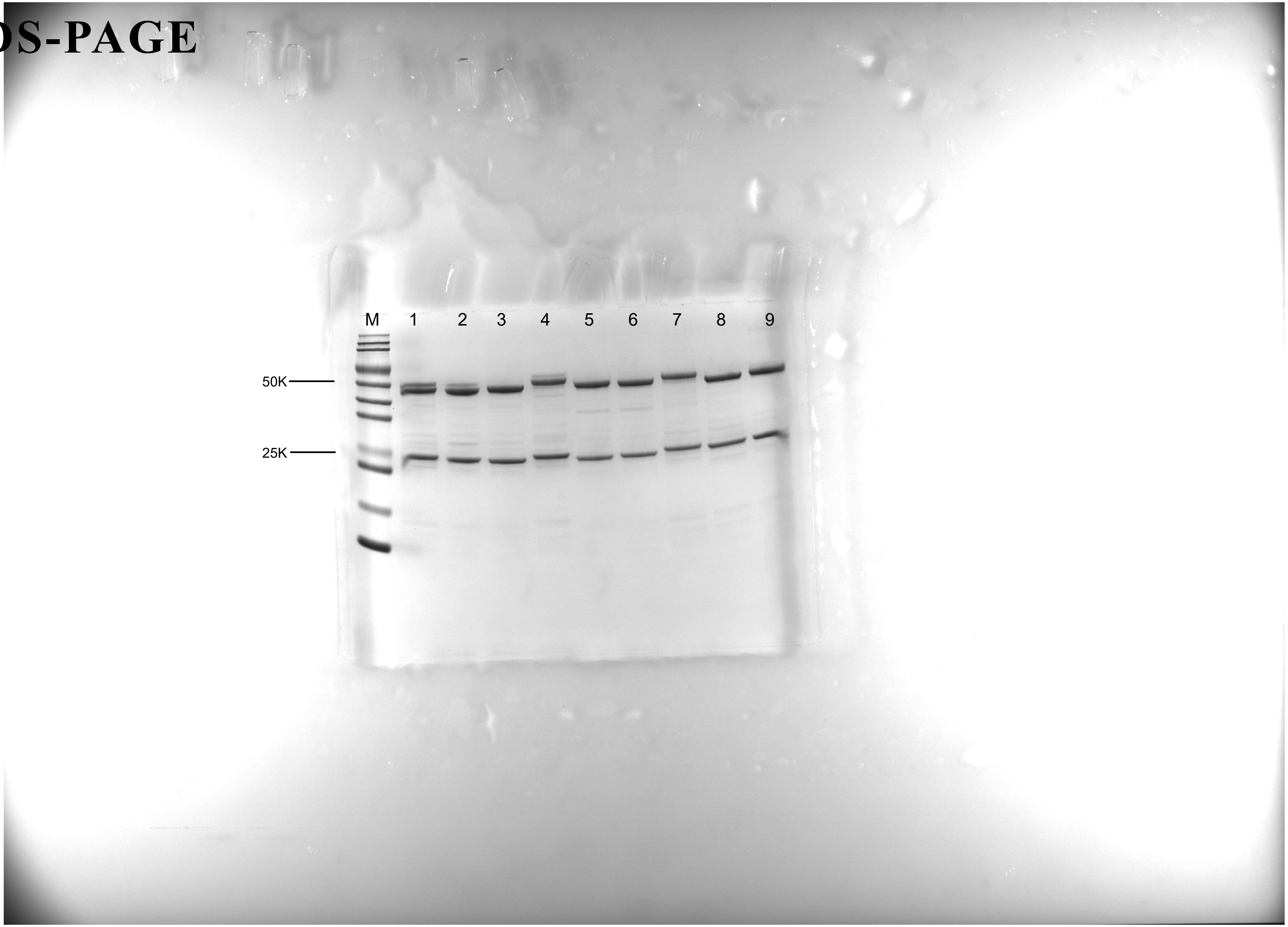

Supplementary figure 1. SDS-PAGE of TREM2 antibodies. Lane M: protein Marker. Lane 1-78: number corresponds to sample name, 3ug, reduced. Lane AL002: 3ug, reduced.

TREM2 mAb SDS-PAGE

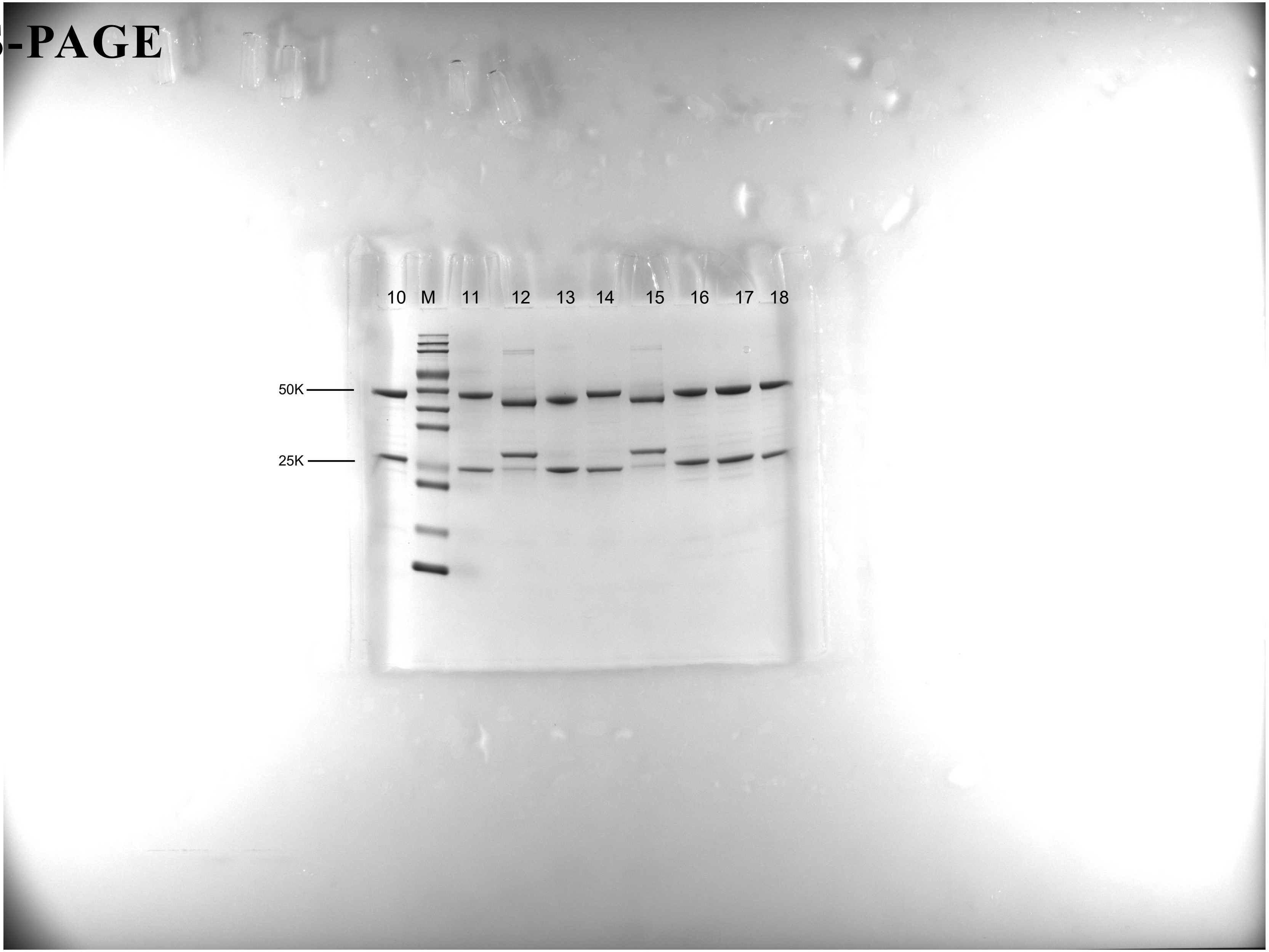

Supplementary figure 1. SDS-PAGE of TREM2 antibodies. Lane M: protein Marker. Lane 1-78: number corresponds to sample name, 3ug, reduced. Lane AL002: 3ug, reduced.

TREM2 mAb SDS-PAGE

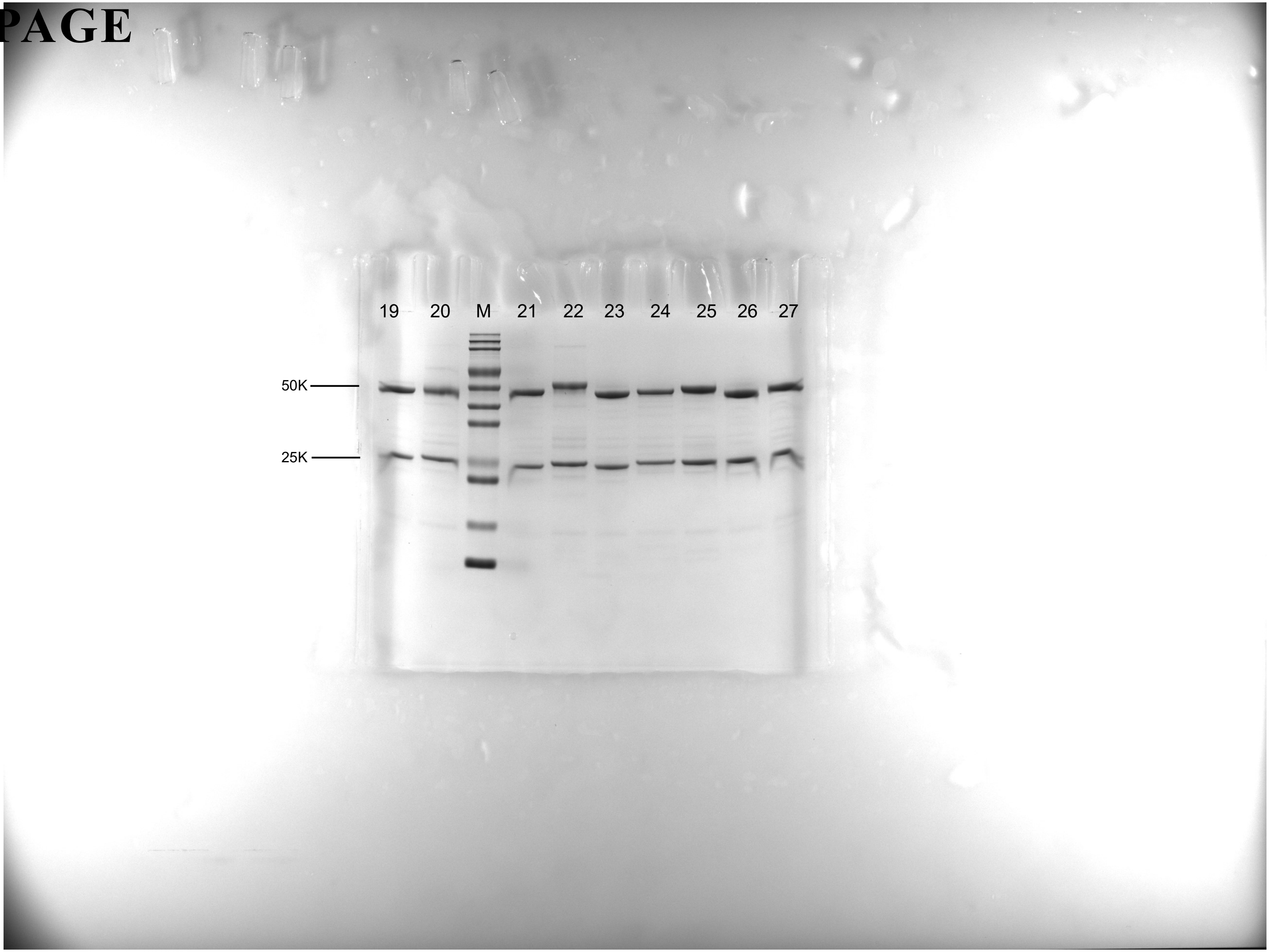

Supplementary figure 1. SDS-PAGE of TREM2 antibodies. Lane M: protein Marker. Lane 1-78: number corresponds to sample name, 3ug, reduced. Lane AL002: 3ug, reduced.

TREM2 mAb SDS-PAGE

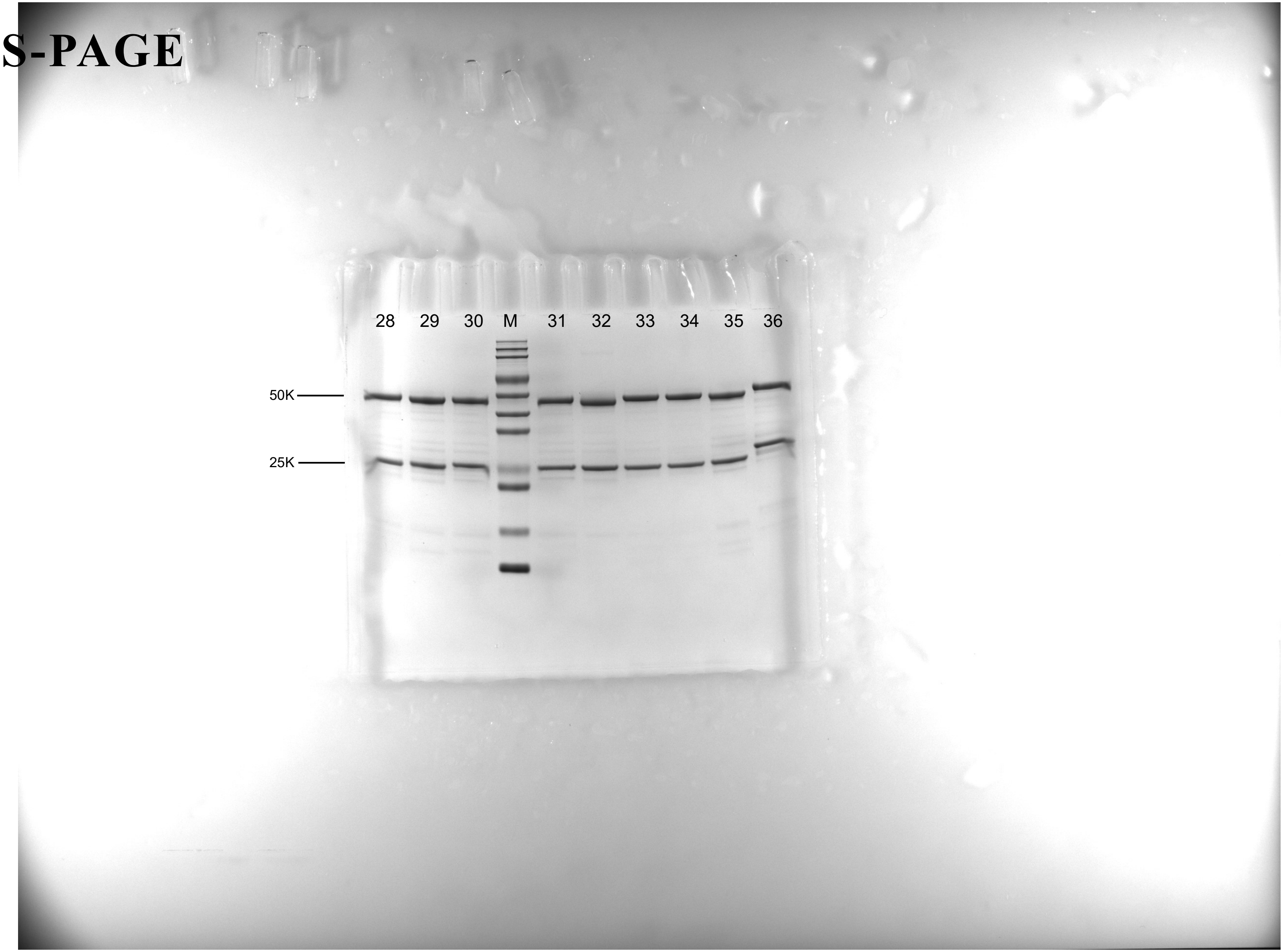

Supplementary figure 1. SDS-PAGE of TREM2 antibodies. Lane M: protein Marker. Lane 1-78: number corresponds to sample name, 3ug, reduced. Lane AL002: 3ug, reduced.

TREM2 mAb SDS-PAGE

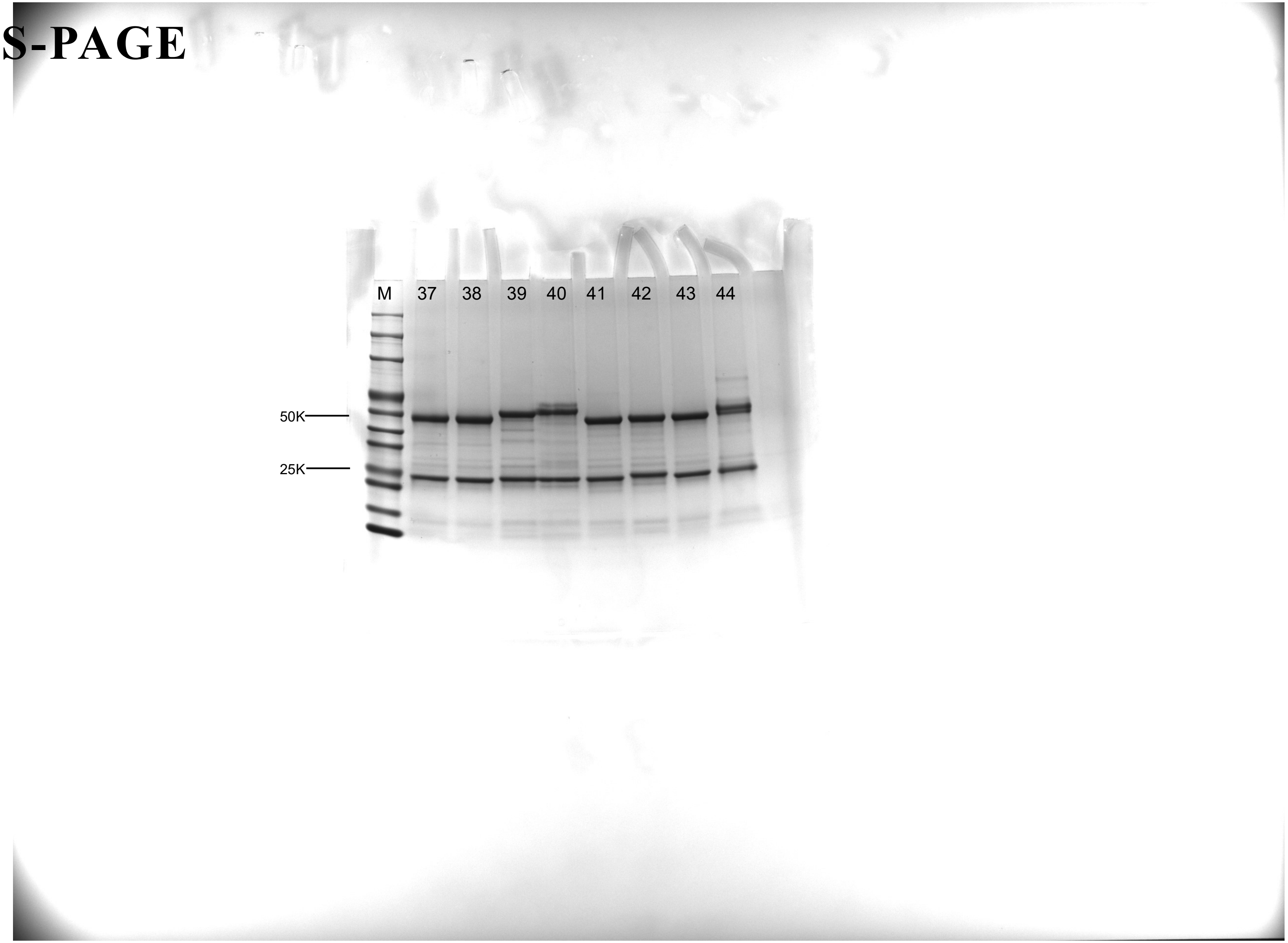

Supplementary figure 1. SDS-PAGE of TREM2 antibodies. Lane M: protein Marker. Lane 1-78: number corresponds to sample name, 3ug, reduced. Lane AL002: 3ug, reduced.

TREM2 mAb SDS-PAGE

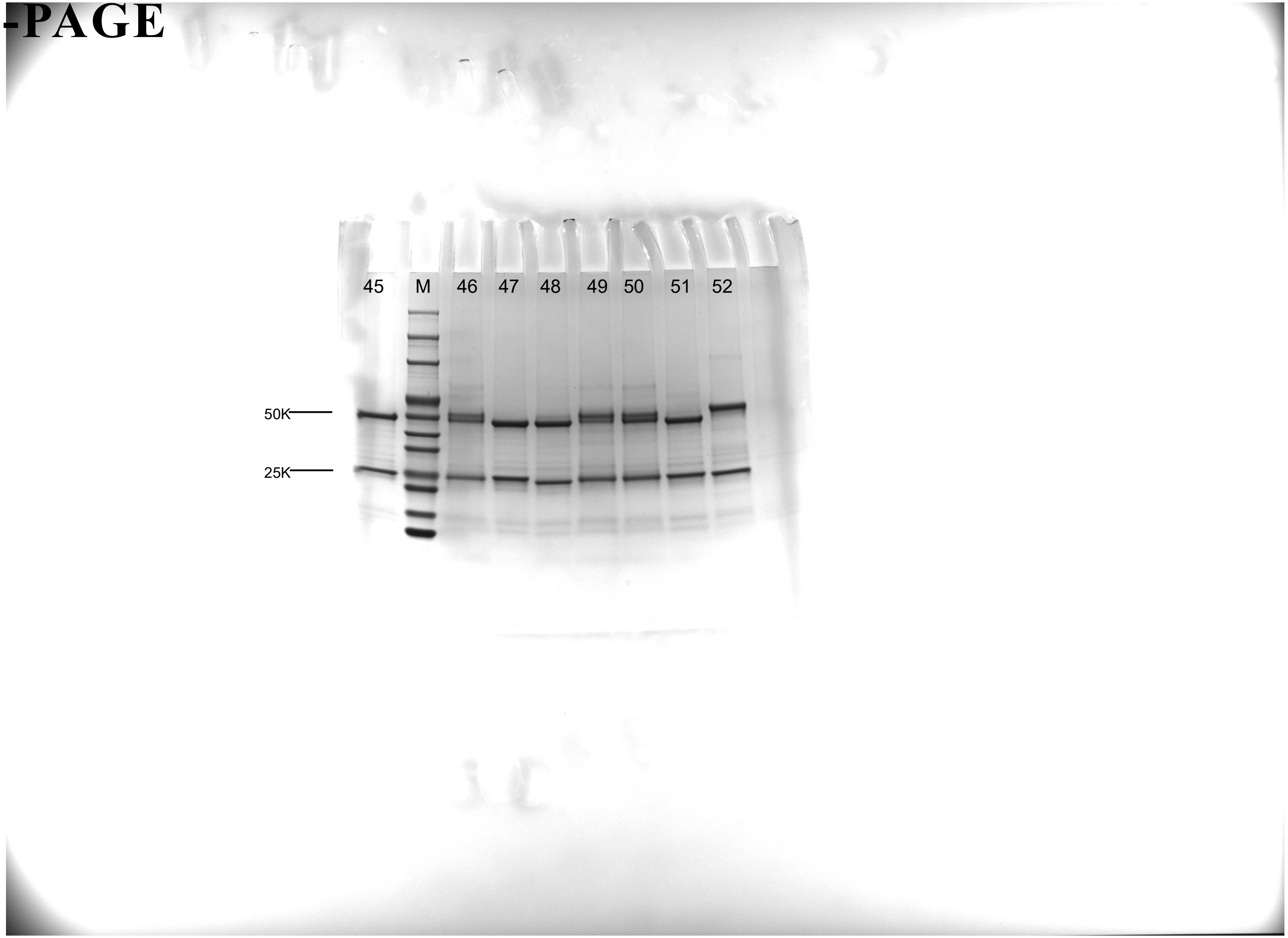

Supplementary figure 1. SDS-PAGE of TREM2 antibodies. Lane M: protein Marker. Lane 1-78: number corresponds to sample name, 3ug, reduced. Lane AL002: 3ug, reduced.

TREM2 mAb SDS-PAGE

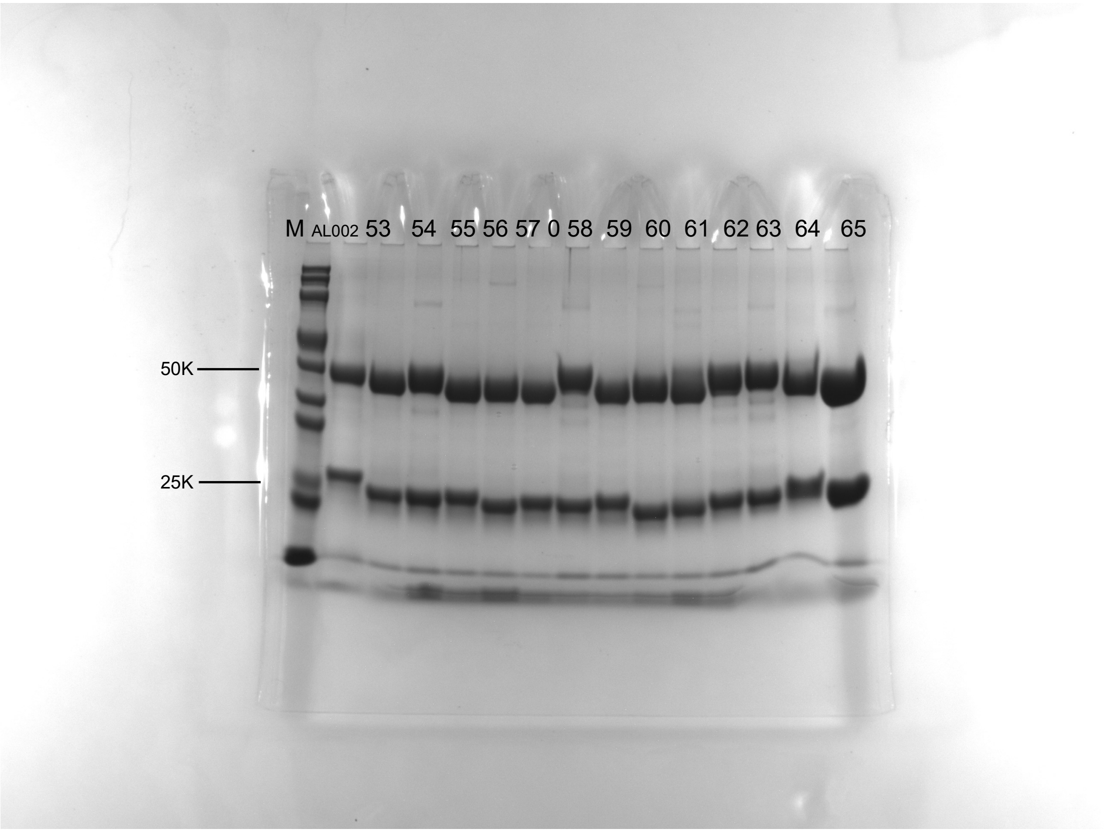

Supplementary figure 1. SDS-PAGE of TREM2 antibodies. Lane M: protein Marker. Lane 1-78: number corresponds to sample name, 3ug, reduced. Lane AL002: 3ug, reduced.

TREM2 mAb SDS-PAGE

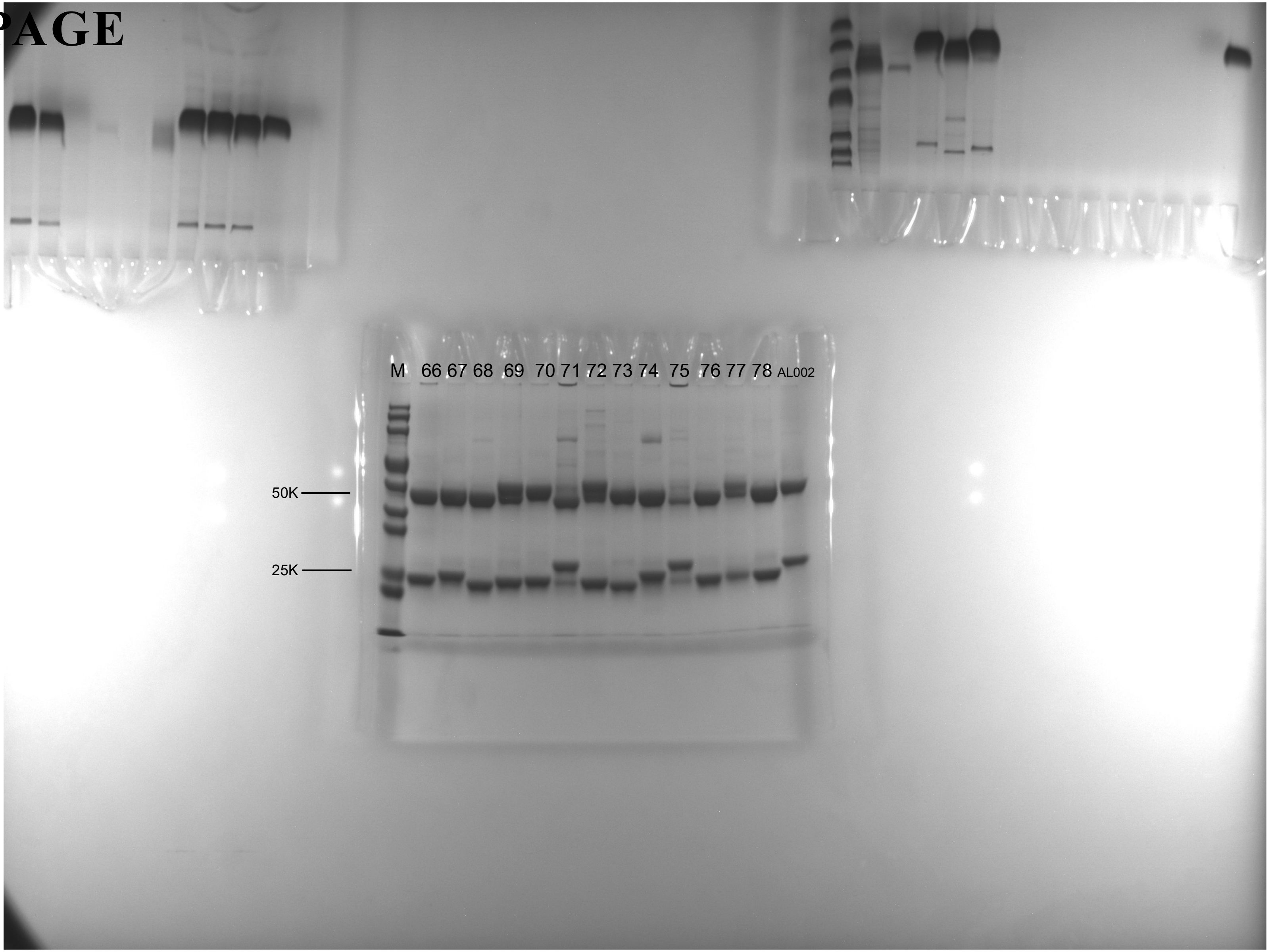

Supplementary figure 1. SDS-PAGE of TREM2 antibodies. Lane M: protein Marker. Lane 1-78: number corresponds to sample name, 3ug, reduced. Lane AL002: 3ug, reduced.

Supplementary table 1. SDS-PAGE of TREM2 antibodies. Summary of purified antibodies. Number corresponds to sample name.

Number	Antibody
**1**	TREM2#1
**2**	TREM2#5
**3**	TREM2#6
**4**	TREM2#7
**5**	TREM2#8
**6**	TREM2#9
**7**	TREM2#10
**8**	TREM2#11
**9**	TREM2#12
**10**	TREM2#13
**11**	TREM2#14
**12**	TREM2#15
**13**	TREM2#16
**14**	TREM2#17
**15**	TREM2#19
**16**	TREM2#20
**17**	TREM2#21
**18**	TREM2#22
**19**	TREM2#23
**20**	TREM2#24
**21**	TREM2#25
**22**	TREM2#26
**23**	TREM2#30
**24**	TREM2#33
**25**	TREM2#34
**26**	TREM2#36
**27**	TREM2#38
**28**	TREM2#46
**29**	TREM2#53
**30**	TREM2#57

Number	Antibody
**31**	TREM2#60
**32**	TREM2#63
**33**	TREM2#65
**34**	TREM2#69
**35**	TREM2#75
**36**	AL002
**37**	TREM2#48
**38**	TREM2#50
**39**	TREM2#52
**40**	TREM2#55
**41**	TREM2#67
**42**	TREM2#70
**43**	TREM2#77
**44**	TREM2#78
**45**	TREM2#10
**46**	TREM2#18
**47**	TREM2#31
**48**	TREM2#35
**49**	TREM2#37
**50**	TREM2#40
**51**	TREM2#43
**52**	TREM2#45
**53**	TREM2#42
**54**	TREM2#44
**55**	TREM2#47
**56**	TREM2#51
**57**	TREM2#54
**58**	TREM2#56
**59**	TREM2#58
**60**	TREM2#61

Number	Antibody
**61**	TREM2#66
**62**	TREM2#68
**63**	TREM2#71
**64**	TREM2#72
**65**	TREM2#74
**66**	TREM2#75
**67**	TREM2#76
**68**	TREM2#79
**69**	TREM2#82
**70**	TREM2#83
**71**	TREM2#84
**72**	TREM2#85
**73**	TREM2#86
**74**	TREM2#87
**75**	TREM2#88
**76**	TREM2#89
**77**	TREM2#90
**78**	TREM2#91
**AL002**	AL002
