# Supplementary material for: Preclinical development of an immunoassay for the detection of TREM2: a new biomarker for Alzheimer’s disease
Source: Sci Rep. 2025 Jul 22;15:26525. doi: 10.1038/s41598-025-09262-x (PMC12280022; doi:10.1038/s41598-025-09262-x)
Supplement: Supplementary file 3 — Supplementary Information 3. [file 41598_2025_9262_MOESM3_ESM.pptx]

## Slide 1
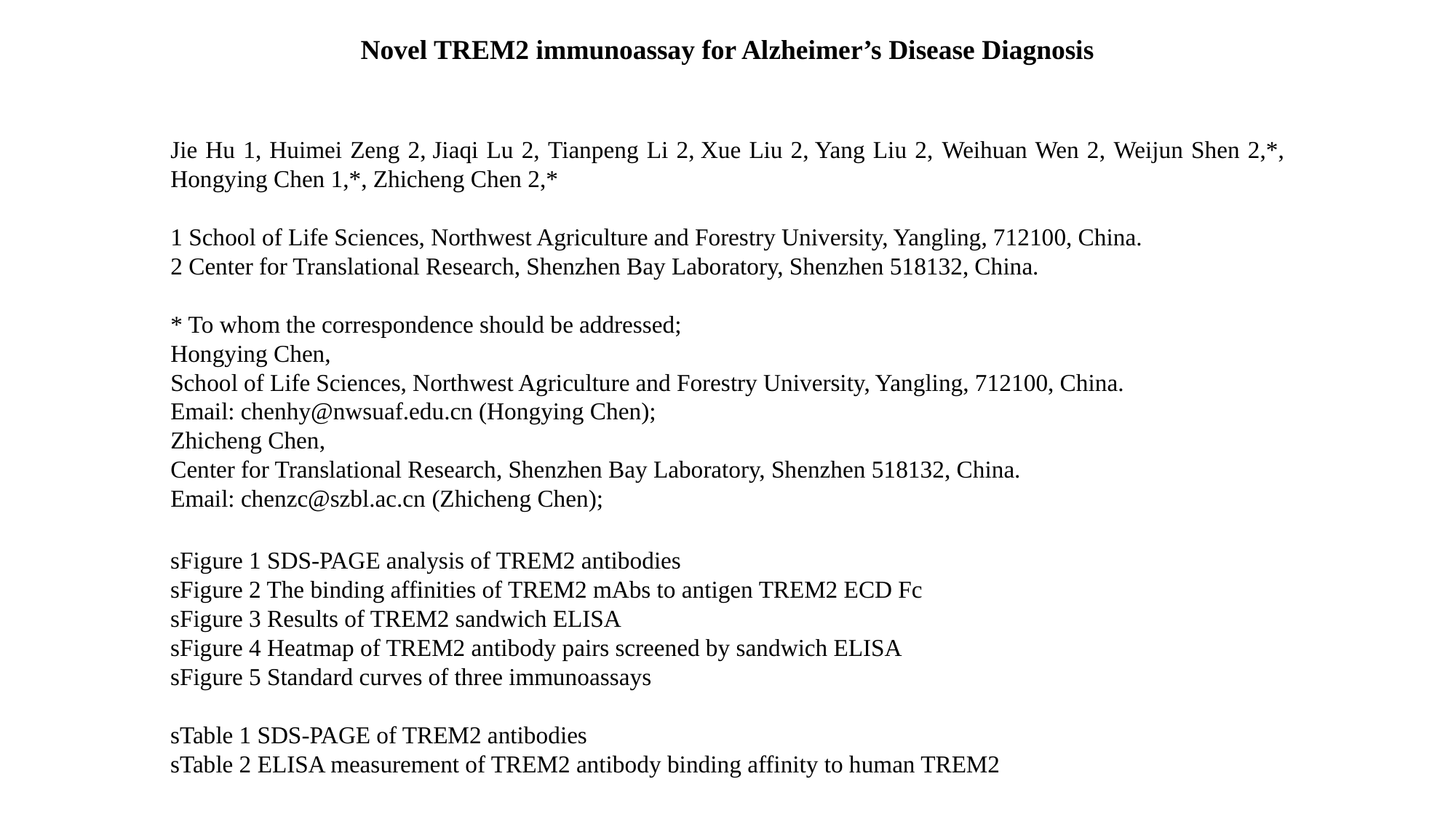

Novel TREM2 immunoassay for Alzheimer’s Disease Diagnosis
Jie Hu 1, Huimei Zeng 2, Jiaqi Lu 2, Tianpeng Li 2, Xue Liu 2, Yang Liu 2, Weihuan Wen 2, Weijun Shen 2,*, Hongying Chen 1,*, Zhicheng Chen 2,*
1 School of Life Sciences, Northwest Agriculture and Forestry University, Yangling, 712100, China.
2 Center for Translational Research, Shenzhen Bay Laboratory, Shenzhen 518132, China.
* To whom the correspondence should be addressed;
Hongying Chen,
School of Life Sciences, Northwest Agriculture and Forestry University, Yangling, 712100, China.
Email: chenhy@nwsuaf.edu.cn (Hongying Chen);
Zhicheng Chen,
Center for Translational Research, Shenzhen Bay Laboratory, Shenzhen 518132, China.
Email: chenzc@szbl.ac.cn (Zhicheng Chen);
sFigure 1 SDS-PAGE analysis of TREM2 antibodies
sFigure 2 The binding affinities of TREM2 mAbs to antigen TREM2 ECD Fc
sFigure 3 Results of TREM2 sandwich ELISA
sFigure 4 Heatmap of TREM2 antibody pairs screened by sandwich ELISA
sFigure 5 Standard curves of three immunoassays
sTable 1 SDS-PAGE of TREM2 antibodies
sTable 2 ELISA measurement of TREM2 antibody binding affinity to human TREM2

## Slide 2
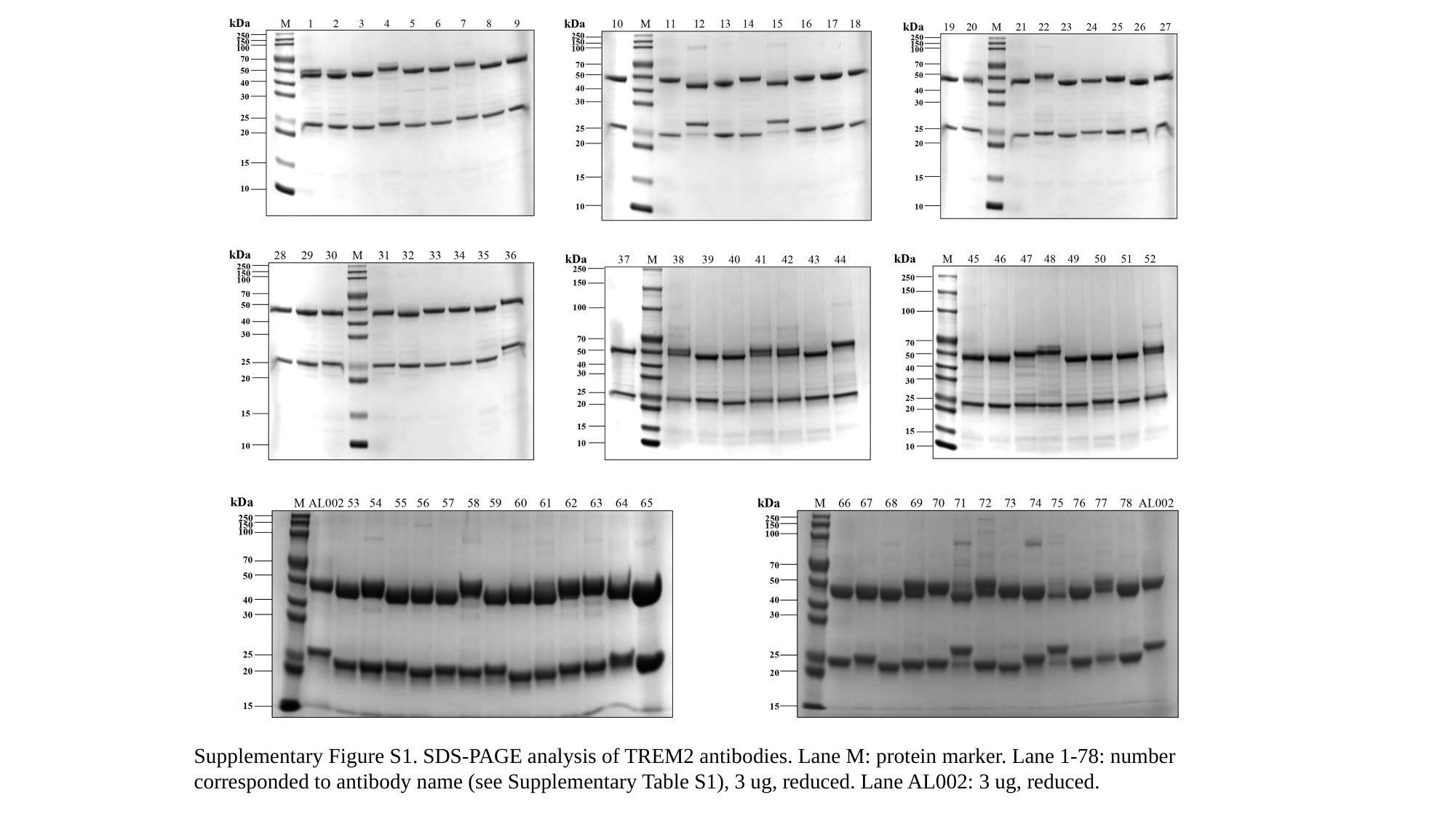

Supplementary Figure S1. SDS-PAGE analysis of TREM2 antibodies. Lane M: protein marker. Lane 1-78: number corresponded to antibody name (see Supplementary Table S1), 3 ug, reduced. Lane AL002: 3 ug, reduced.

## Slide 3
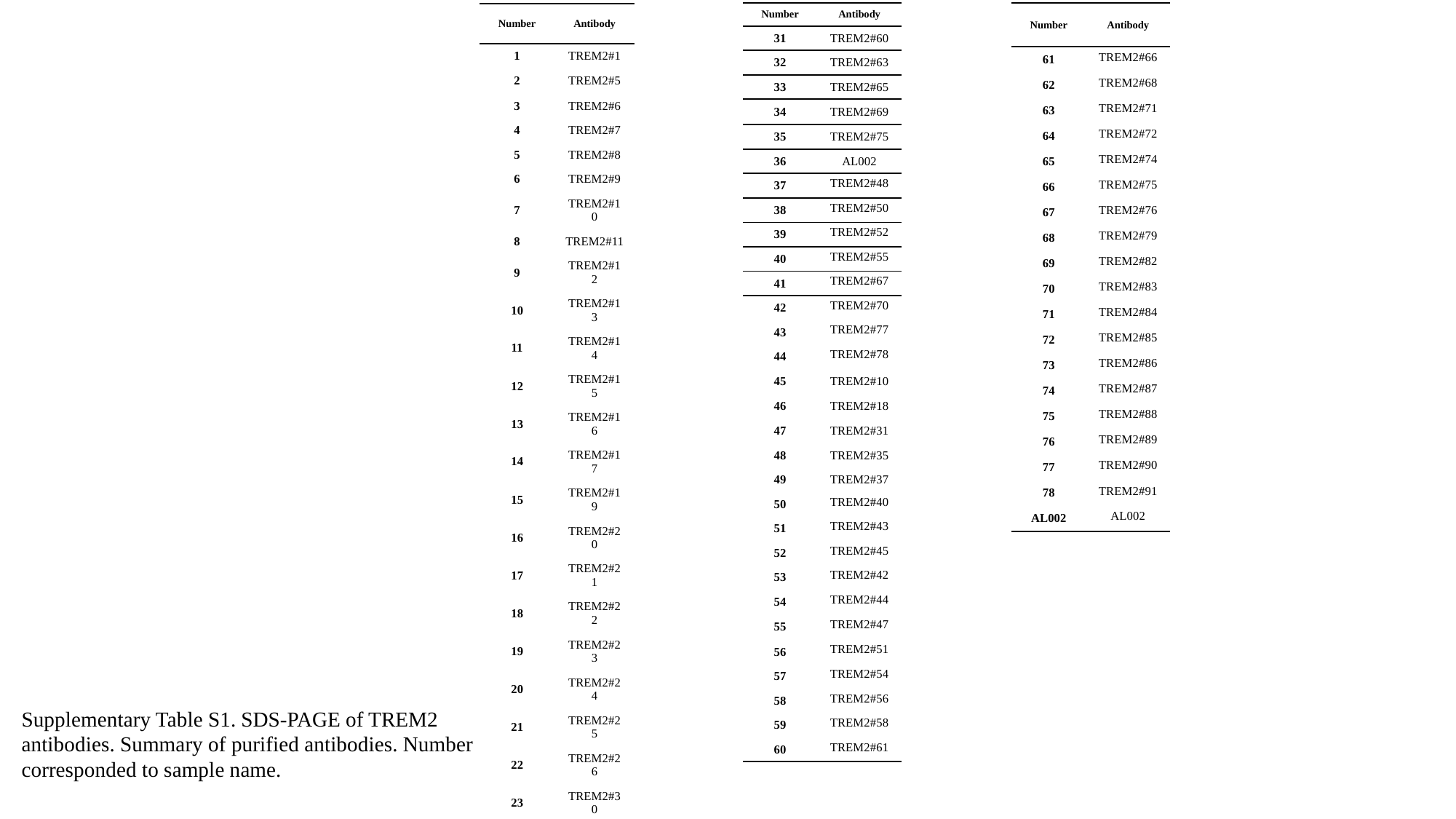

| Number | Antibody |
| --- | --- |
| 31 | TREM2#60 |
| 32 | TREM2#63 |
| 33 | TREM2#65 |
| 34 | TREM2#69 |
| 35 | TREM2#75 |
| 36 | AL002 |
| 37 | TREM2#48 |
| 38 | TREM2#50 |
| 39 | TREM2#52 |
| 40 | TREM2#55 |
| 41 | TREM2#67 |
| 42 | TREM2#70 |
| 43 | TREM2#77 |
| 44 | TREM2#78 |
| 45 | TREM2#10 |
| 46 | TREM2#18 |
| 47 | TREM2#31 |
| 48 | TREM2#35 |
| 49 | TREM2#37 |
| 50 | TREM2#40 |
| 51 | TREM2#43 |
| 52 | TREM2#45 |
| 53 | TREM2#42 |
| 54 | TREM2#44 |
| 55 | TREM2#47 |
| 56 | TREM2#51 |
| 57 | TREM2#54 |
| 58 | TREM2#56 |
| 59 | TREM2#58 |
| 60 | TREM2#61 |
| Number | Antibody |
| --- | --- |
| 61 | TREM2#66 |
| 62 | TREM2#68 |
| 63 | TREM2#71 |
| 64 | TREM2#72 |
| 65 | TREM2#74 |
| 66 | TREM2#75 |
| 67 | TREM2#76 |
| 68 | TREM2#79 |
| 69 | TREM2#82 |
| 70 | TREM2#83 |
| 71 | TREM2#84 |
| 72 | TREM2#85 |
| 73 | TREM2#86 |
| 74 | TREM2#87 |
| 75 | TREM2#88 |
| 76 | TREM2#89 |
| 77 | TREM2#90 |
| 78 | TREM2#91 |
| AL002 | AL002 |
| Number | Antibody |
| --- | --- |
| 1 | TREM2#1 |
| 2 | TREM2#5 |
| 3 | TREM2#6 |
| 4 | TREM2#7 |
| 5 | TREM2#8 |
| 6 | TREM2#9 |
| 7 | TREM2#10 |
| 8 | TREM2#11 |
| 9 | TREM2#12 |
| 10 | TREM2#13 |
| 11 | TREM2#14 |
| 12 | TREM2#15 |
| 13 | TREM2#16 |
| 14 | TREM2#17 |
| 15 | TREM2#19 |
| 16 | TREM2#20 |
| 17 | TREM2#21 |
| 18 | TREM2#22 |
| 19 | TREM2#23 |
| 20 | TREM2#24 |
| 21 | TREM2#25 |
| 22 | TREM2#26 |
| 23 | TREM2#30 |
| 24 | TREM2#33 |
| 25 | TREM2#34 |
| 26 | TREM2#36 |
| 27 | TREM2#38 |
| 28 | TREM2#46 |
| 29 | TREM2#53 |
| 30 | TREM2#57 |
Supplementary Table S1. SDS-PAGE of TREM2 antibodies. Summary of purified antibodies. Number corresponded to sample name.

## Slide 4
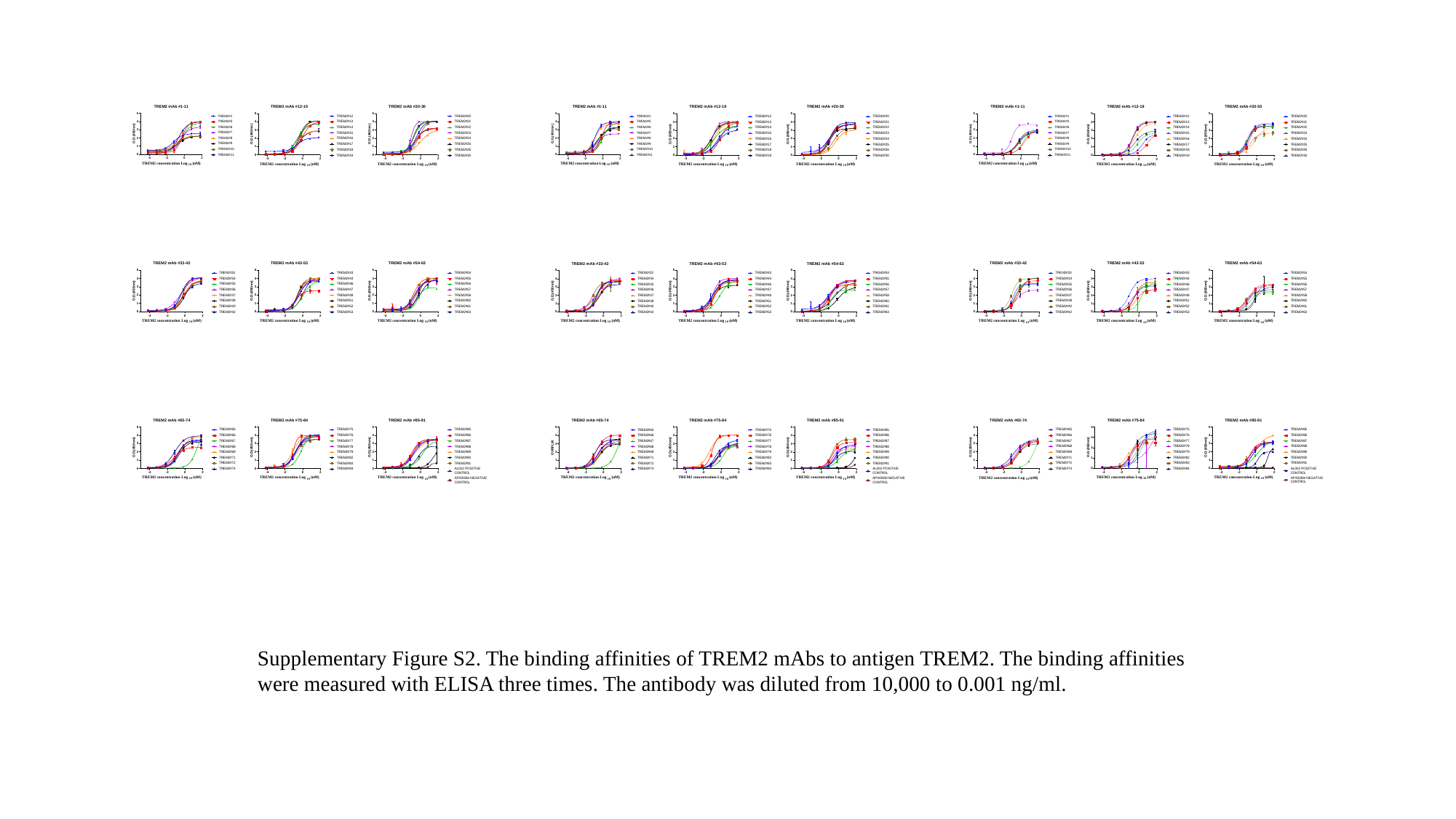

Supplementary Figure S2. The binding affinities of TREM2 mAbs to antigen TREM2. The binding affinities were measured with ELISA three times. The antibody was diluted from 10,000 to 0.001 ng/ml.

## Slide 5
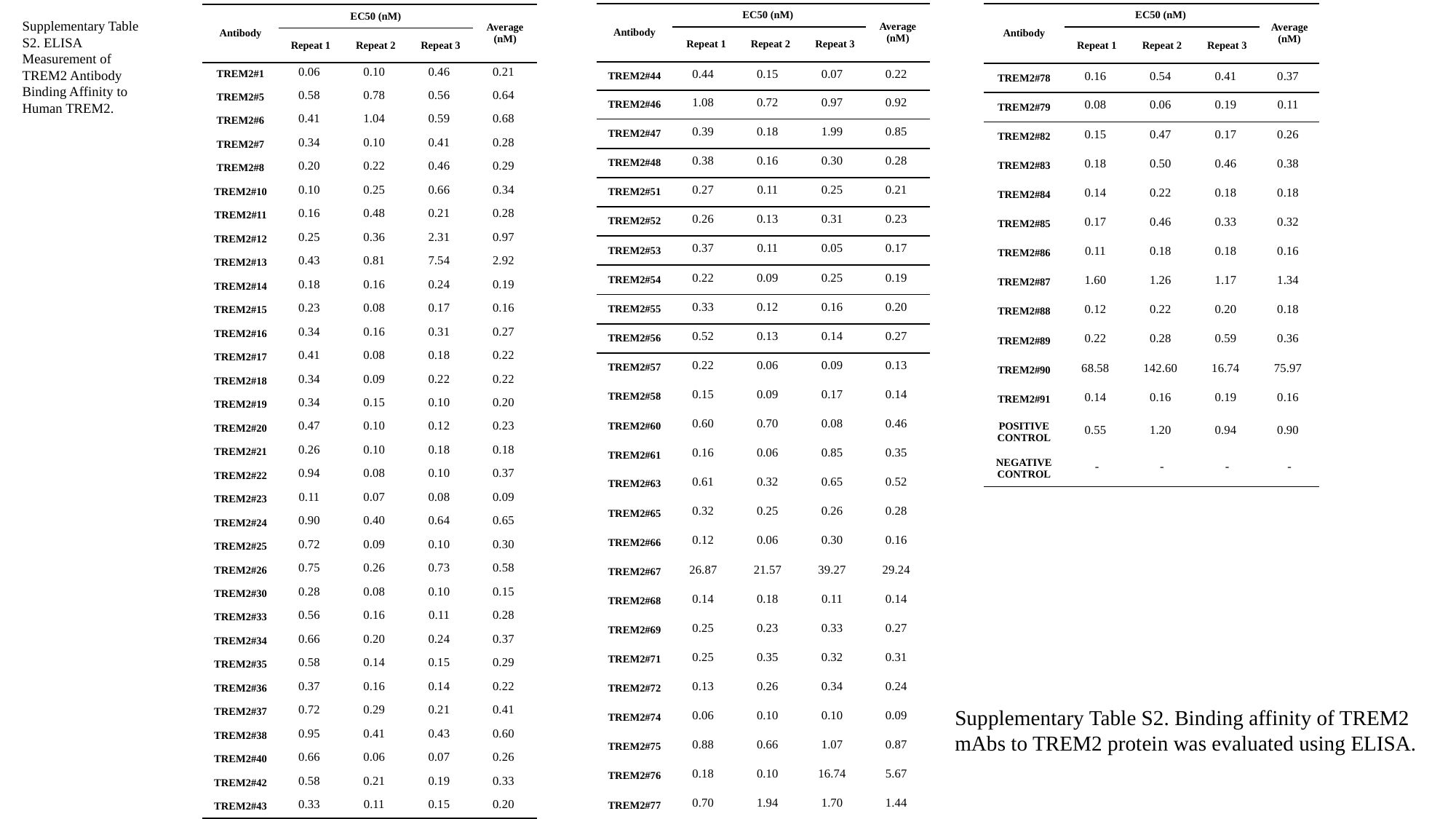

| Antibody | EC50 (nM) | | | Average (nM) |
| --- | --- | --- | --- | --- |
| | Repeat 1 | Repeat 2 | Repeat 3 | |
| TREM2#44 | 0.44 | 0.15 | 0.07 | 0.22 |
| TREM2#46 | 1.08 | 0.72 | 0.97 | 0.92 |
| TREM2#47 | 0.39 | 0.18 | 1.99 | 0.85 |
| TREM2#48 | 0.38 | 0.16 | 0.30 | 0.28 |
| TREM2#51 | 0.27 | 0.11 | 0.25 | 0.21 |
| TREM2#52 | 0.26 | 0.13 | 0.31 | 0.23 |
| TREM2#53 | 0.37 | 0.11 | 0.05 | 0.17 |
| TREM2#54 | 0.22 | 0.09 | 0.25 | 0.19 |
| TREM2#55 | 0.33 | 0.12 | 0.16 | 0.20 |
| TREM2#56 | 0.52 | 0.13 | 0.14 | 0.27 |
| TREM2#57 | 0.22 | 0.06 | 0.09 | 0.13 |
| TREM2#58 | 0.15 | 0.09 | 0.17 | 0.14 |
| TREM2#60 | 0.60 | 0.70 | 0.08 | 0.46 |
| TREM2#61 | 0.16 | 0.06 | 0.85 | 0.35 |
| TREM2#63 | 0.61 | 0.32 | 0.65 | 0.52 |
| TREM2#65 | 0.32 | 0.25 | 0.26 | 0.28 |
| TREM2#66 | 0.12 | 0.06 | 0.30 | 0.16 |
| TREM2#67 | 26.87 | 21.57 | 39.27 | 29.24 |
| TREM2#68 | 0.14 | 0.18 | 0.11 | 0.14 |
| TREM2#69 | 0.25 | 0.23 | 0.33 | 0.27 |
| TREM2#71 | 0.25 | 0.35 | 0.32 | 0.31 |
| TREM2#72 | 0.13 | 0.26 | 0.34 | 0.24 |
| TREM2#74 | 0.06 | 0.10 | 0.10 | 0.09 |
| TREM2#75 | 0.88 | 0.66 | 1.07 | 0.87 |
| TREM2#76 | 0.18 | 0.10 | 16.74 | 5.67 |
| TREM2#77 | 0.70 | 1.94 | 1.70 | 1.44 |
| Antibody | EC50 (nM) | | | Average (nM) |
| --- | --- | --- | --- | --- |
| | Repeat 1 | Repeat 2 | Repeat 3 | |
| TREM2#78 | 0.16 | 0.54 | 0.41 | 0.37 |
| TREM2#79 | 0.08 | 0.06 | 0.19 | 0.11 |
| TREM2#82 | 0.15 | 0.47 | 0.17 | 0.26 |
| TREM2#83 | 0.18 | 0.50 | 0.46 | 0.38 |
| TREM2#84 | 0.14 | 0.22 | 0.18 | 0.18 |
| TREM2#85 | 0.17 | 0.46 | 0.33 | 0.32 |
| TREM2#86 | 0.11 | 0.18 | 0.18 | 0.16 |
| TREM2#87 | 1.60 | 1.26 | 1.17 | 1.34 |
| TREM2#88 | 0.12 | 0.22 | 0.20 | 0.18 |
| TREM2#89 | 0.22 | 0.28 | 0.59 | 0.36 |
| TREM2#90 | 68.58 | 142.60 | 16.74 | 75.97 |
| TREM2#91 | 0.14 | 0.16 | 0.19 | 0.16 |
| POSITIVE CONTROL | 0.55 | 1.20 | 0.94 | 0.90 |
| NEGATIVE CONTROL | - | - | - | - |
| Antibody | EC50 (nM) | | | Average (nM) |
| --- | --- | --- | --- | --- |
| | Repeat 1 | Repeat 2 | Repeat 3 | |
| TREM2#1 | 0.06 | 0.10 | 0.46 | 0.21 |
| TREM2#5 | 0.58 | 0.78 | 0.56 | 0.64 |
| TREM2#6 | 0.41 | 1.04 | 0.59 | 0.68 |
| TREM2#7 | 0.34 | 0.10 | 0.41 | 0.28 |
| TREM2#8 | 0.20 | 0.22 | 0.46 | 0.29 |
| TREM2#10 | 0.10 | 0.25 | 0.66 | 0.34 |
| TREM2#11 | 0.16 | 0.48 | 0.21 | 0.28 |
| TREM2#12 | 0.25 | 0.36 | 2.31 | 0.97 |
| TREM2#13 | 0.43 | 0.81 | 7.54 | 2.92 |
| TREM2#14 | 0.18 | 0.16 | 0.24 | 0.19 |
| TREM2#15 | 0.23 | 0.08 | 0.17 | 0.16 |
| TREM2#16 | 0.34 | 0.16 | 0.31 | 0.27 |
| TREM2#17 | 0.41 | 0.08 | 0.18 | 0.22 |
| TREM2#18 | 0.34 | 0.09 | 0.22 | 0.22 |
| TREM2#19 | 0.34 | 0.15 | 0.10 | 0.20 |
| TREM2#20 | 0.47 | 0.10 | 0.12 | 0.23 |
| TREM2#21 | 0.26 | 0.10 | 0.18 | 0.18 |
| TREM2#22 | 0.94 | 0.08 | 0.10 | 0.37 |
| TREM2#23 | 0.11 | 0.07 | 0.08 | 0.09 |
| TREM2#24 | 0.90 | 0.40 | 0.64 | 0.65 |
| TREM2#25 | 0.72 | 0.09 | 0.10 | 0.30 |
| TREM2#26 | 0.75 | 0.26 | 0.73 | 0.58 |
| TREM2#30 | 0.28 | 0.08 | 0.10 | 0.15 |
| TREM2#33 | 0.56 | 0.16 | 0.11 | 0.28 |
| TREM2#34 | 0.66 | 0.20 | 0.24 | 0.37 |
| TREM2#35 | 0.58 | 0.14 | 0.15 | 0.29 |
| TREM2#36 | 0.37 | 0.16 | 0.14 | 0.22 |
| TREM2#37 | 0.72 | 0.29 | 0.21 | 0.41 |
| TREM2#38 | 0.95 | 0.41 | 0.43 | 0.60 |
| TREM2#40 | 0.66 | 0.06 | 0.07 | 0.26 |
| TREM2#42 | 0.58 | 0.21 | 0.19 | 0.33 |
| TREM2#43 | 0.33 | 0.11 | 0.15 | 0.20 |
Supplementary Table S2. ELISA Measurement of TREM2 Antibody Binding Affinity to Human TREM2.
Supplementary Table S2. Binding affinity of TREM2 mAbs to TREM2 protein was evaluated using ELISA.

## Slide 6
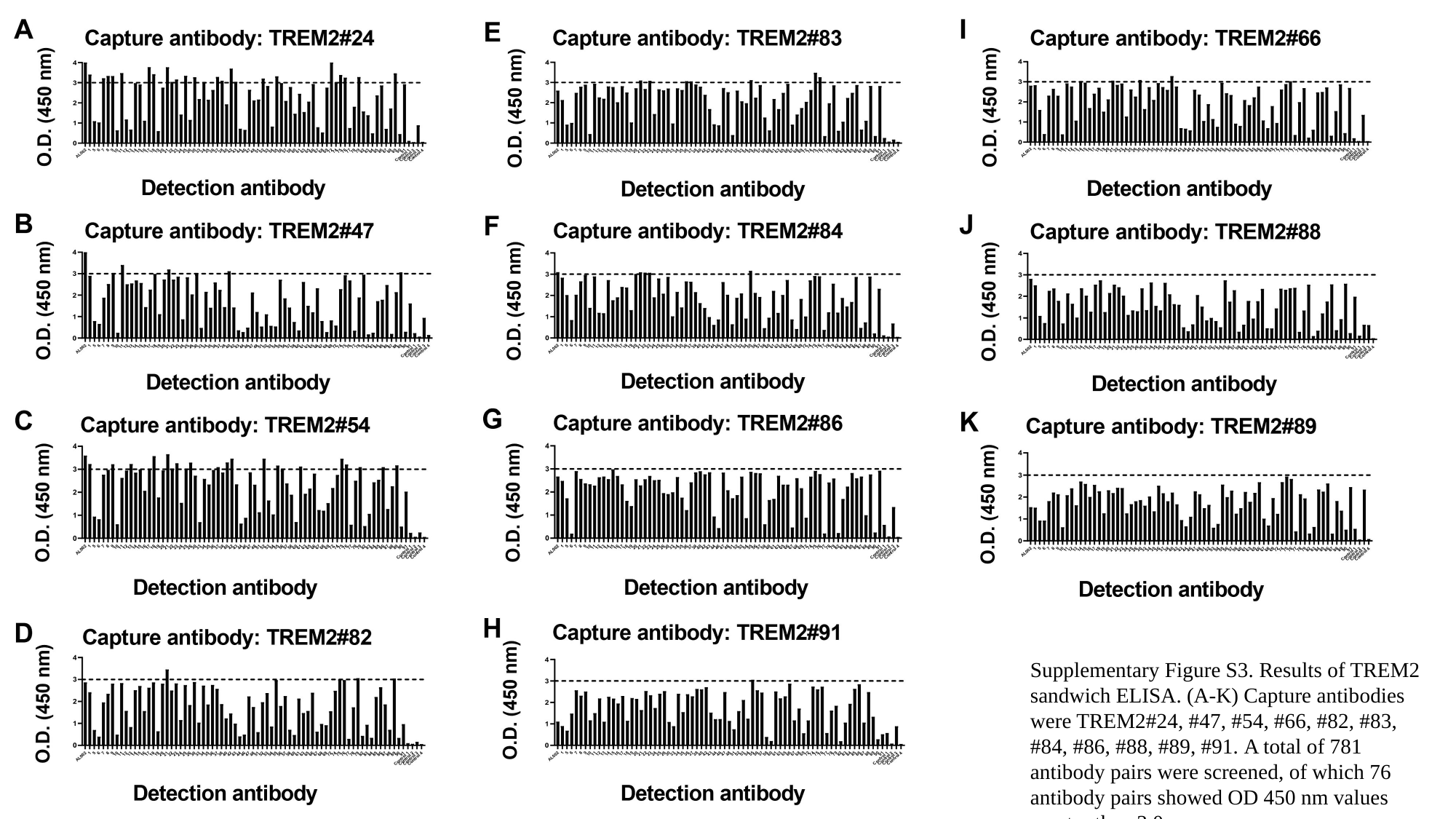

Supplementary Figure S3. Results of TREM2 sandwich ELISA. (A-K) Capture antibodies were TREM2#24, #47, #54, #66, #82, #83, #84, #86, #88, #89, #91. A total of 781 antibody pairs were screened, of which 76 antibody pairs showed OD 450 nm values greater than 3.0.

## Slide 7
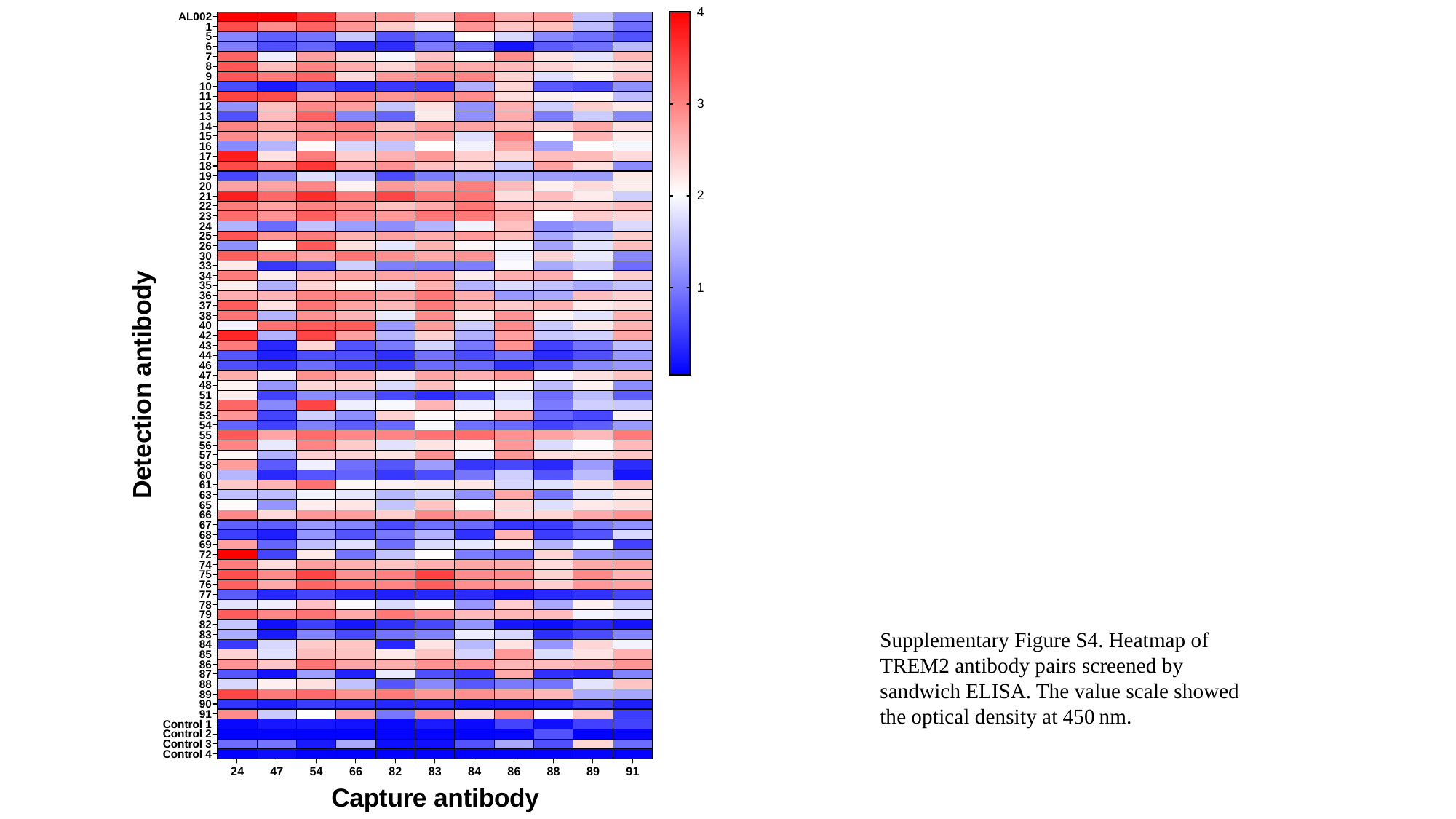

Supplementary Figure S4. Heatmap of TREM2 antibody pairs screened by sandwich ELISA. The value scale showed the optical density at 450 nm.

## Slide 8
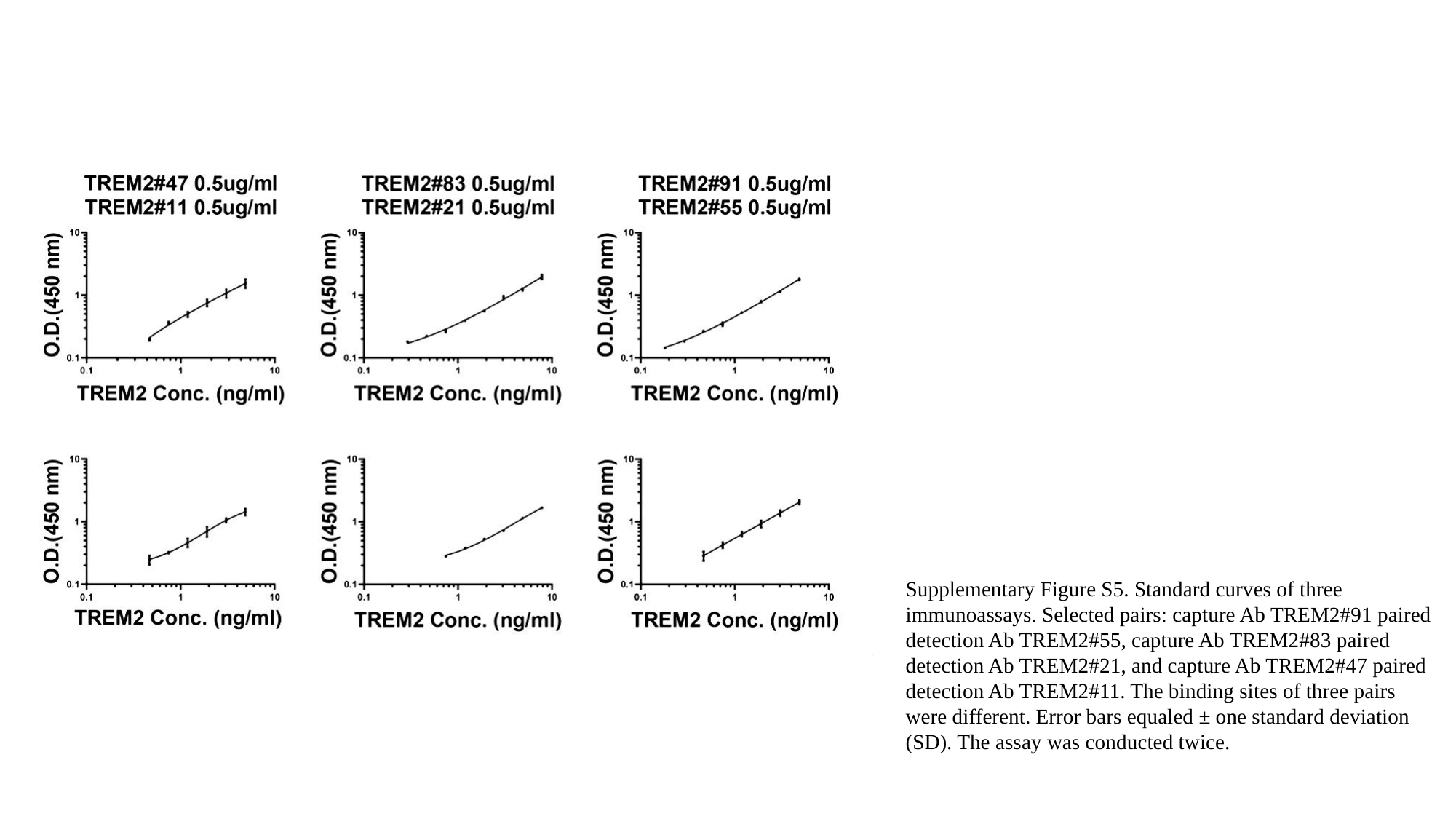

Supplementary Figure S5. Standard curves of three immunoassays. Selected pairs: capture Ab TREM2#91 paired detection Ab TREM2#55, capture Ab TREM2#83 paired detection Ab TREM2#21, and capture Ab TREM2#47 paired detection Ab TREM2#11. The binding sites of three pairs were different. Error bars equaled ± one standard deviation (SD). The assay was conducted twice.
